# Incidence of self-reported tuberculosis treatment with community-wide universal testing and treatment for HIV and tuberculosis screening in Zambia and South Africa: A planned analysis of the HPTN 071 (PopART) cluster-randomised trial

**DOI:** 10.1371/journal.pmed.1004393

**Published:** 2024-05-31

**Authors:** L. Telisinghe, S. Floyd, D. MacLeod, A. Schaap, R. Dunbar, J. Bwalya, N. Bell-Mandla, E. Piwowar-Manning, D. Donnell, K. Shaunaube, P. Bock, S. Fidler, R. J. Hayes, H. M. Ayles, on behalf of the HPTN 071 (PopART) study team

**Affiliations:** 1 Department of Clinical Research, Faculty of Infectious and Tropical Diseases, London School of Hygiene and Tropical Medicine, London, United Kingdom; 2 Zambart, Lusaka, Zambia; 3 Department of Infectious Disease Epidemiology, Faculty of Epidemiology and Population Health, London School of Hygiene and Tropical Medicine, London, United Kingdom; 4 The Desmond Tutu Tuberculosis Centre, Department of Paediatrics and Child Health, Faculty of Medicine and Health Sciences, Stellenbosch University, Cape Town, South Africa; 5 Johns Hopkins University School of Medicine, Baltimore, Maryland, United States of America; 6 The Fred Hutchinson Cancer Research Center, Seattle, Washington, United States of America; 7 Imperial College, London, United Kingdom; 8 National Institute for Health Research, Imperial Biomedical Research Centre, London, United Kingdom; University of Southampton, UNITED KINGDOM

## Abstract

**Background:**

HIV is a potent risk factor for tuberculosis (TB). Therefore, community-wide universal testing and treatment for HIV (UTT) could contribute to TB control, but evidence for this is limited. Community-wide TB screening can decrease population-level TB prevalence. Combining UTT with TB screening could therefore significantly impact TB control in sub-Saharan Africa, but to our knowledge there is no evidence for this combined approach.

**Methods and findings:**

HPTN 071 (PopART) was a community-randomised trial conducted between November 2013 to July 2018; 21 Zambian and South African communities (with a total population of approximately 1 million individuals) were randomised to arms A (community-wide UTT and TB screening), B (community-wide universal HIV testing with treatment following national guidelines and TB screening), or C (standard-of-care). In a cohort of randomly selected adults (18 to 44 years) enrolled between 2013 and 2015 from all 21 communities (total size 38,474; 27,139 [71%] female; 8,004 [21%] HIV positive) and followed-up annually for 36 months to measure the population-level impact of the interventions, data on self-reported TB treatment in the previous 12 months (self-reported TB) were collected by trained research assistants and recorded using a structured questionnaire at each study visit. In this prespecified analysis of the trial, self-reported TB incidence rates were measured by calendar year between 2014 and 2017/2018. A *p*-value ≤0.05 on hypothesis testing was defined as reaching statistical significance. Between January 2014 and July 2018, 38,287 individuals were followed-up: 494 self-reported TB during 104,877 person-years. Overall incidence rates were similar across all arms in 2014 and 2015 (0.33 to 0.46/100 person-years). In 2016 incidence rates were lower in arm A compared to C overall (adjusted rate ratio [aRR] 0.48 [95% confidence interval (95% CI) 0.28 to 0.81; *p* = 0.01]), with statistical significance reached. In 2017/2018, while incidence rates were lower in arm A compared to C, statistical significance was not reached (aRR 0.58 [95% CI 0.27 to 1.22; *p* = 0.13]). Among people living with HIV (PLHIV) incidence rates were lower in arm A compared to C in 2016 (RR 0.56 [95% CI 0.29 to 1.08; *p* = 0.08]) and 2017/2018 (RR 0.50 [95% CI 0.26 to 0.95; *p* = 0.04]); statistical significance was only reached in 2017/2018. Incidence rates in arms B and C were similar, overall and among PLHIV. Among HIV–negative individuals, there were too few events for cross-arm comparisons. Study limitations include the use of self-report which may have been subject to under-reporting, limited covariate adjustment due to the small number of events, and high losses to follow-up over time.

**Conclusions:**

In this study, community-wide UTT and TB screening resulted in substantially lower TB incidence among PLHIV at population-level, compared to standard-of-care, with statistical significance reached in the final study year. There was also some evidence this translated to a decrease in self-reported TB incidence overall in the population. Reduction in arm A but not B suggests UTT drove the observed effect. Our data support the role of UTT in TB control, in addition to HIV control, in high TB/HIV burden settings.

**Trial registration:**

ClinicalTrials.gov: NCT01900977.

## Abstract 2

Lily Telisinghe and co-workers study self-reported tuberculosis incidence among communities in Zambia and South Africa in the PopART trial of universal HIV testing and treatment and tuberculosis screening.

## Author summary

**Why was this study done?:**

- Tuberculosis (TB) is a leading cause of sickness and death worldwide. In sub-Saharan Africa, TB is mainly driven by the HIV-epidemic.
- Between 2013 and 2018, the HPTN 071 cluster-randomised trial was conducted in 21 Zambian and South African communities. There were 3 study arms: (1) arm A which received universal testing and treatment for HIV (UTT) and TB screening; (2) arm B which received universal HIV testing (with antiretroviral therapy (ART) according to national guidelines) and TB screening; and (3) arm C the control.
- As part of the trial, a cohort of 38,474 adults aged 18 to 44 years were enrolled from all communities at the start and followed-up annually over 36 months.

**What did the researchers do and find?:**

- All cohort members were asked if they had been started on TB treatment in the last 12 months (self-reported TB), at each annual visit (maximum of 4 visits). We investigated the effect of the interventions on self-reported TB incidence.
- We found a decrease in self-reported TB incidence among people living with HIV in arm A compared to arm C. There was also some evidence this translated to a decrease in self-reported TB incidence overall in the population in arm A compared to arm C.
- Self-reported TB incidence was similar in arms B and C, overall in the population and among people living with HIV.
- We could not determine the effect of the interventions on self-reported TB incidence among those who were HIV negative, due to the small number of events.

**What do these findings mean?:**

- The decrease in self-reported TB incidence in arm A (which received community-wide UTT and TB screening) but not arm B (which received community-wide HIV testing with ART according to national guidelines and TB screening) suggests the UTT component of the intervention drove the changes observed in arm A.
- Our data support the role of UTT in TB control in sub-Saharan Africa.

## Background

Tuberculosis (TB) is a leading infectious cause of morbidity and mortality worldwide [1]. Sub-Saharan Africa has some of the highest TB incidence and mortality rates, which are mainly driven by the generalised HIV epidemic [1,2]. While current TB control measures have gradually decreased population-level TB incidence in the region, steeper reductions are needed to meet the ambitious World Health Organization (WHO) End TB Strategy milestones and targets: compared to 2015, a 50% and 90% reduction in TB incidence by 2025 and 2035, respectively [1]. However, the best approach to control TB in sub-Saharan Africa is unknown.

At the individual-level, antiretroviral therapy (ART) decreases the risk of incident TB among people living with HIV (PLHIV) who take ART [2]. ART roll-out started in ~2004 in sub-Saharan Africa, with the CD4^+^ T-lymphocyte threshold for ART initiation and therefore ART coverage increasing over time. In 2015, WHO recommended “universal ART” (i.e., starting ART irrespective of CD4^+^ T-lymphocyte count) for PLHIV [3]. Several observational studies have tried to determine the potential for ART to control TB, both in the total population and among all PLHIV (those in and not in HIV/ART-care), where the individual-level effect of ART among PLHIV taking ART may not necessarily translate to the population-level impact needed for TB control. Several routine programmes have observed decreases in TB notification rates/diagnoses coincident with routine ART scale-up over time [4–15]. Three further observational studies using different study designs and outcomes, found an association between increasing ART coverage under routine programmatic conditions and decreases in population-level measures of TB [16–18]. While it is plausible that ART use may in part explain these observations, it is not possible to conclude based on these observational findings alone, that ART can control TB in sub-Saharan Africa.

In sub-Saharan Africa, the HIV testing landscape is also changing. The Joint United Nations Programme for HIV/AIDS (UNAIDS) targets, include 95% of PLHIV knowing their status and 95% of those diagnosed receiving ART [19]. To meet these goals, countries must go beyond providing ART only to those seeking HIV care. One approach is universal HIV testing (i.e., repeated HIV testing of whole populations), combined with linkage-to-care and support to initiate universal ART, along with treatment adherence support. This intervention is called universal testing and treatment for HIV (or UTT). Several trials have shown that UTT can be implemented effectively, help meet UNAIDS targets, and decrease population-level HIV incidence [20]. Mathematical modelling also predicts that with annual HIV testing and universal ART, the HIV-associated TB incidence could decrease by approximately 50% once full coverage is reached [21]. But to date, these predictions have not been robustly investigated.

To control TB, WHO also recommends systematic TB screening in general populations with high TB prevalence [22]. TB screening aims to, irrespective of HIV-status, identify and treat people with infectious undiagnosed TB early, decreasing the background TB transmission risk [22]. Therefore, combining UTT and TB screening could achieve large, rapid, and sustained decreases in population-level TB incidence, but empirical data supporting this combined approach are lacking.

HPTN 071 (PopART) was a cluster-randomised HIV treatment as prevention trial conducted in Zambia and the Western Cape of South Africa; the intervention package included community-wide UTT and systematic TB screening [23,24]. The primary outcome of the trial, HIV incidence, was measured in a cohort of adults aged 18 to 44 years who were followed-up for 36 months. The effect of the HPTN 071 (PopART) interventions on HIV incidence and other key secondary outcomes have already been published [24–26]. During follow-up, cohort members were also asked if they initiated TB treatment (self-reported TB). Here, we investigated the effect of the intervention on self-reported TB incidence at population-level, a planned secondary analysis of the trial.

## Methods

Ethical approval for the trial was obtained from the London School of Hygiene and Tropical Medicine UK, University of Zambia, and Stellenbosch University South Africa. The trial design has been described in detail elsewhere and is briefly summarised here (also see S1 CONSORT Checklist) [23–25,27].

### Population

Twenty-one urban and peri-urban communities (12 Zambian and 9 South African; total population approximately 1 million), with high HIV prevalence (10% to 25%), high TB case notification rates (≥400/100,000 population), and population ≥20,000 were purposively selected. A community was the catchment population of a health centre and communities were geographically distinct. Communities were matched into triplets (groups of 3 communities), based on geography and HIV prevalence, giving 4 Zambian and 3 South African triplets. The communities in each triplet were then randomised to one of 3 study arms (2 intervention arms [A and B] and a standard-of-care arm [C]), using restricted randomisation to ensure balance across arms by population size, baseline ART coverage, and HIV prevalence.

### Intervention

In arms A and B, between November 2013 and June 2015, a door-to-door, community-wide, HIV/TB prevention intervention was delivered by trained community health workers (S1–S3 Appendix). Between July 2015 and December 2017, 2 further intervention rounds were delivered. At each intervention round, all households in the intervention communities were visited and offered the study intervention. In both arms universal HIV testing using rapid tests was offered, with linkage-to-care if HIV–positive. ART initiation was universal in arm A from 2013. In arm B, ART initiation followed national guidelines, which switched to universal treatment in 2016 (Zambia in April and South Africa in October). In both arms, a symptom questionnaire (any one of cough ≥2 weeks, night sweats or unintentional weight loss ≥1.5 kg in the preceding month) was used to screen for TB. If symptomatic, sputum was collected and tested according to national guidelines (using Xpert MTB/RIF and smear). If sputum positive, individuals were linked to TB treatment. Arm C, the control, received the standard-of-care, through routine services. This included mainly passive TB case finding for people attending health centres. Provider-initiated TB symptom screening was conducted for PLHIV attending ART care. HIV counselling and testing was available at health centres for those seeking HIV care services and those identified as having presumptive TB.

### Outcome

To measure trial outcomes, a population cohort (PC) was established between November 2013 and March 2015 (S3 Appendix). One adult aged 18 to 44 years was randomly selected from a random sample of households in all 21 communities at baseline (called PC0). The cohort was followed-up at 12, 24, and 36 months (called PC12, PC24, and PC36, respectively). PC36 ended in July 2018.

Our primary outcome was self-reported TB measured in a closed cohort enrolled at PC0. At each PC-visit (PC0 to PC36), trained research assistants administered a structured questionnaire using electronic data capture devices. All PC-participants were asked: (1) if they had been told they had TB in the preceding 12 months; (2) if yes, did they start TB treatment (specified as the preceding 12 months in PC12-36, but not at PC0); and (3) if yes, the month and year of treatment start (Table 1). At PC0, self-reported TB was defined as starting TB treatment in the 14 months before the PC-visit (duration calculated using month/year of PC-visit and treatment start date). A 14-month eligibility period (rather than 12 months) was used to allow for errors in recalling months. For PC12–36, in addition to this, individuals unable to recall treatment start month/year were also included in the case definition, as the question specified if treatment was started “in the last 12 months.” At each PC-visit, blood was collected and tested in the laboratory to determine HIV-status.

**Table 1:** Number and proportion meeting the case definition of self-reported TB treatment by PC visit, in the cohort enrolled at PC0 (*N* = 38,474) from all 21 HPTN 071 (PopART) communities.

		PC0	PC12	PC24	PC36
		*N* = 38,474	*N* = 25,290	*N* = 21,678	*N* = 20,422
Self-reported being told they had TB AND starting TB treatment^‡^	Yes	361/38,474 (0.94%)	164/25,290 (0.65%)	177/21,678 (0.82%)	110/20,422 (0.54%)
Duration between visit date and MM/YYYY of TB treatment start^⎰^^Ŧ^	Missing^¶^	67/361 (18.56%)	21/164 (12.80%)	17/177 (9.60%)	12/110 (10.91%)
	≤14 months*	279/361 (77.29%)	121/164 (73.78%)	143/177 (80.80%)	93/110 (84.55%)
	>14 months*	15/361 (4.16%)	22/164 (13.41%)	17/177 (9.60%)	5/110 (4.54%)
Total meeting the case definition of self-reported TB treatment	Yes^†^ ^¥^	279/38,474 (0.73%)	142/25,290 (0.56%)	160/21,678 (0.74%)	105/20,422 (0.51%)
Total meeting case definition by country	Zambia	114/19,724 (0.58%)	37/12,331 (0.30%)	46/10,927 (0.42%)	34/10,945 (0.31%)
	South Africa	165/18,750 (0.88%)	105/12,959 (0.81%)	114/10,751 (1.06%)	71/9,477 (0.75%)
Total meeting case definition by HIV-status^$^	Negative^ϕ^	79/29,130 (0.27%)	43/17,669 (0.24%)	67/15,294 (0.44%)	43/15,111 (0.28%)
	Positive	192/8,004 (2.40%)	84/5,086 (1.65%)	75/4,579 (1.64%)	60/4,758 (1.26%)
	Not determined	8/1,340 (0.60%)	15/2,535 (0.59%)	18/1,805 (1.00%)	2/553 (0.36%)

^‡^**Question asked:** “in the last 12 months, have you been told that you have TB” with response options of yes, no and do not know. If response was yes, **Question asked:** at PC0 “have you started TB treatment” and at PC12-36 “have you started TB treatment in the last 12 months” with response options of yes, no and do not know.

^⎰^If response was yes to “in the last 12 months, have you been told that you have TB” AND yes to “have you started TB treatment/have you started TB treatment in the last 12 months”, **Question asked:** “When did you start TB treatment? Please give the month and year?”.

^Ŧ^Denominator is number of people self-reporting being told they have TB in the last 12 months and starting TB treatment.

^¶^Unable to calculate duration due to missing month and year of TB treatment start.

*Number of months between self-reported TB treatment start month and year, and, visit date.

^†^At PC0, self-reported TB treatment included individuals reporting TB treatment start in the 14 months before the PC-visit. For PC12–36, self-reported TB treatment was defined as individuals reporting TB treatment start in the 14 months before the PC-visit AND individuals who were unable to recall treatment start month/year (as the question at PC12–36 specified if treatment was started “in the last 12 months”).

^¥^Proportion of participants meeting the self-reported TB case definition who could recall the month and year of TB treatment start only (overall, by country, by HIV status) shown in S9 Appendix.

^$^HIV-status based on laboratory testing.

^ϕ^Among those HIV–negative, there were no events in multiple communities when data were disaggregated by community (at PC0 there were no events in 4 communities, at PC12 there were no events in 6 communities, at PC24 there were no events in 3 communities and at PC36 there were no events in 7 communities).

TB, tuberculosis; PC, population cohort; MM/YYYY, month and year of TB treatment start.

### Statistical methods (also see S4 Appendix)

Two approaches were used to analyse data: cohort (primary analysis) and cross-sectional (secondary analysis).

#### Cohort analysis

Because self-reported TB was determined over the 14 months before each PC-visit, for each PC-participant, an observation start date 14 months before each PC-visit was generated, representing the date from which their observation time for each PC-visit began. To determine incidence, the analysis used the first self-reported TB event observed for each participant. Where month and year of treatment start was known, the day was imputed as 15. Where the date was unknown (only approximately 10% of those self-reporting TB between PC12–36, where PC-participants who could not recall the month/year of TB treatment start were also included in the case definition; Table 1), it was imputed as the mid-point between the PC-visit with self-reported TB and the preceding PC-visit if there were 2 consecutive PC-visits, or 7 months before the PC-visit with self-reported TB if no consecutive PC-visits. Entry to the cohort was the observation start date generated 14 months before the PC0-visit. Time-at-risk was calculated from the PC0 observation start date until self-reported TB or the last PC-visit, whichever came first. Not all PC-participants were seen at each PC-visit. Therefore, there were gaps in observation time between PC-visits and the observation start date of the subsequent PC-visit, during which outcome status was unknown. These gaps in follow-up were not included in the time-at-risk.

TB screening, by diagnosing and linking people with TB to treatment, should initially increase self-reported TB [28]. When TB transmission and therefore incidence falls, self-reported TB should decrease [28]. UTT should decrease self-reported TB among PLHIV and overall [15]. Therefore, to investigate patterns and exclude initial rises in self-reported TB due to TB screening, time-at-risk was split into calendar year and analysed by year, starting in 2014; the first year during which the intervention was rolled out. As follow-up in 2018 was only 6 months, 2017/2018 was analysed as one calendar period.

HIV-status at each PC-visit was assumed to be the HIV-status for the whole year in which the PC-visit took place. Where there were discordant HIV-results (positive and negative) for a year (because 2 PC-visits occurred in 1 year), HIV-status was assumed to be positive. Where HIV-status was unknown in the year before a positive result, sensitivity analysis explored assuming HIV-status was positive (as the observation period for a PC-visit extends into the preceding year) or negative in the preceding year.

The rate of self-reported TB (overall and by HIV-status) was calculated for each community, each year; 0.5 was added to the numerator if no individuals self-reported TB. The geometric means of these rates were then compared between study arms.

#### Cross-sectional analysis

Each PC-visit was treated as an independent cross-sectional sample, giving 4 independent cross-sectional samples. All PC-participants seen at a PC-visit, contributed to the analysis for that visit. HIV-status at the PC-visit was assumed to be the HIV-status during the 14-month eligibility period used to measure the outcome. The proportion self-reporting TB (overall and by HIV-status) at each PC-visit was calculated for each community; 0.5 was added to the numerator if no individuals self-reported TB. The geometric means of these proportions were then compared between study arms.

#### Rate ratios (RR)/prevalence ratios (PR)

Arms A and C, and arms B and C were compared; overall and for PLHIV. Cross-arm comparisons were not conducted for HIV–negative individuals due to the very small number of events when data were disaggregated by community and calendar period/PC-visit. Statistical inferences used the recommended two-stage approach, adjusting for covariates at Stage-1 [29]. Stage-1 used Poisson regression for the cohort analysis and logistic regression for the cross-sectional analysis, to compute the expected number of individuals with self-reported TB, assuming no intervention effect. Due to the small number of events during later calendar years/PC-visits, the total population analysis included triplet and HIV-status as covariates (without adjusting for age and sex). Analyses for PLHIV included triplet alone. At Stage-2, a two-way analysis of variance was conducted on the log(observed/expected number self-reporting TB) in each community, with matched triplet and study arm as factors, to generate the overall RR (cohort analysis) and PR (cross-sectional analysis) and 95% confidence intervals (CIs) for cross-arm comparisons. A *p*-value ≤0.05 on hypothesis testing was defined as reaching statistical significance. All analyses were undertaken in Stata version-15 (Stata Corporation, Texas, United States of America).

## Results

### PC-participant characteristics and self-reported TB at PC-visits

In total, 38,474 individuals were enrolled at PC0 (S5 and S6 Appendix). The majority (27,139/38,474 [71%]) were female, 15,225/38,474 (40%) were aged 18 to 24 years, and 8,004/38,474 (21%) were PLHIV. Baseline characteristics were similar across study arms at PC0. Of those enrolled at PC0, 27,948/38,474 (73%) were seen at least once during follow-up. The characteristics of those only seen at PC0 (i.e., had no follow-up PC-visits) and those seen at least once during follow-up were similar (S7 Appendix). By PC-visit, 25,290/38,474 (66%), 21,678/38,474 (56%), and 20,422/38,474 (53%), were seen at PC12, PC24, and PC36, respectively; the proportions seen at each PC-visit were similar across study arms. Despite losses to follow-up at each PC-visit, the characteristics of those seen (overall and among PLHIV [S8 Appendix]) were similar across study arms. Further, the characteristics of those seen were similar to those who were not seen (S7 Appendix). The self-reported TB case definition (Table 1 and S9 Appendix) was met by 279/38,474 (0.73%) at PC0, 142/25,290 (0.56%) at PC12, 160/21,678 (0.74%) at PC24, and 105/20,422 (0.51%) at PC36. The 686 events at all PC-visits were from 628 PC-participants; 573/628 (91%) only self-reported TB at 1 PC-visit. The proportion self-reporting TB was higher in South Africa than in Zambia and among PLHIV than those HIV–negative. Among PLHIV, the proportion self-reporting TB fell from 2.4% in PC0 to 1.3% in PC36.

### Cohort analysis

**Fig 1:**
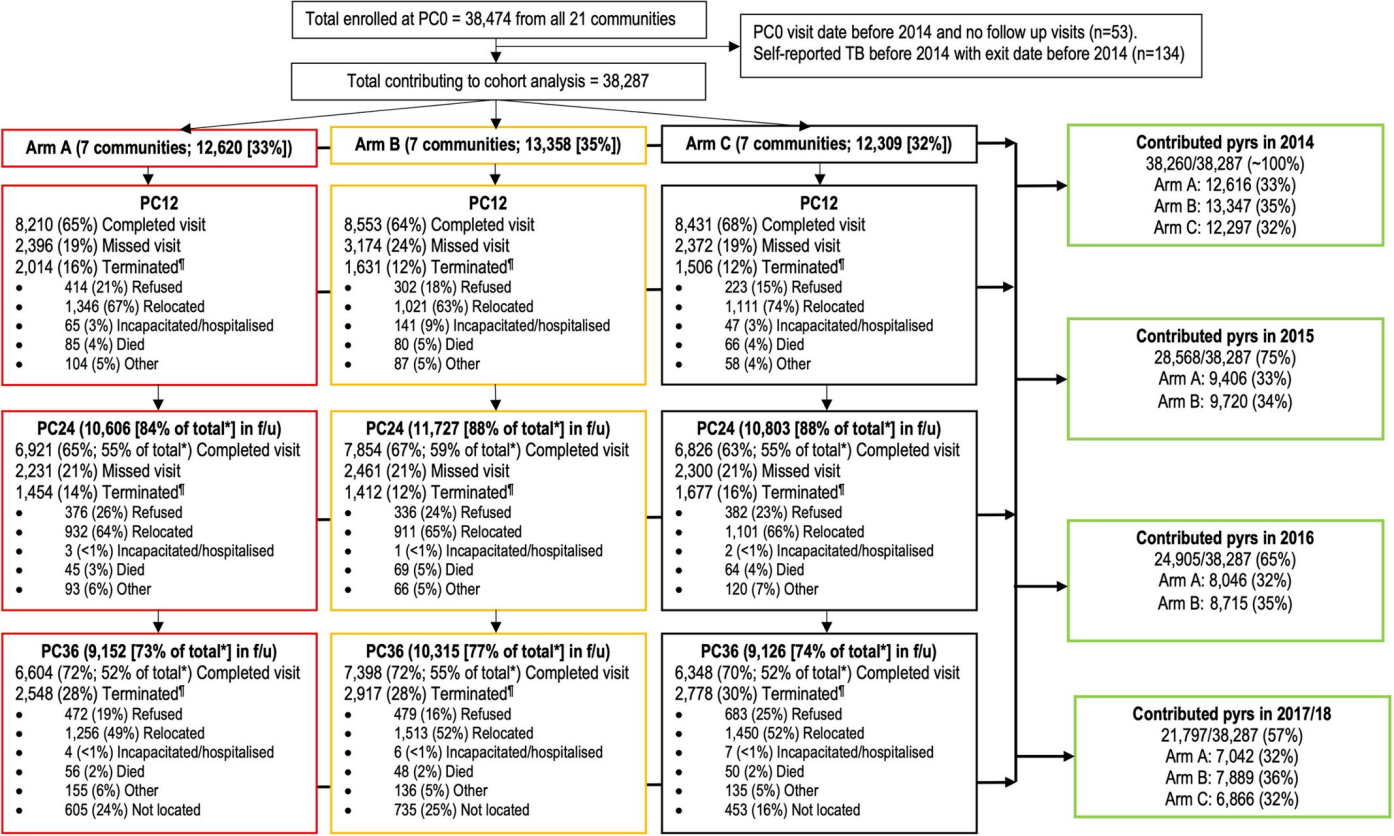
Consort flow diagram showing the PC participants from all 21 HPTN 071 (PopART) communities that contributed person time to the cohort analysis. PC, population cohort; f/u, follow-up; pyrs, person years; ^¶^1 person with unknown termination reason at PC12; 42 people with unknown termination reason at PC24 (5 [<1%] in arm A; 29 [2%] in arm B; and 8 [<1%] in arm C); *denominator the total enrolled in that arm at PC0.

To measure incidence rates, the first self-reported TB event for each participant was used. Between January 2014 and July 2018, 38,287/38,474 (>99%) provided person-time, with 494 events observed. The proportion contributing person-time each year and their characteristics were similar across study arms, both overall (Fig 1 and Table 2 and S10 Appendix) and among PLHIV (S8 Appendix).

**Table 2:** Characteristics of PC participants contributing person time to the cohort analysis from all 21 HPTN 071 (PopART) communities in 2014 (the first study year), by study arm.

			2014	
		A	B	C
**Total *N***		12,616^¶^ (33%)*	13,347^¶^ (35%)*	12,297^¶^ (32%)*
Country	Zambia	6,465 (51%)	6,389 (48%)	6,736 (55%)
	SA	6,151 (49%)	6,958 (52%)	5,561 (45%)
Community HIV prevalence^×^		18%	19%	19%
Community size^Ø^		34,273	40,535	31,068
Sex	Male	3,578 (28%)	3,890 (29%)	3,655 (30%)
	Female	9,004 (72%)	9,417 (71%)	8,583 (70%)
Age/years^‡^	18–24	5,045 (40%)	5,162 (39%)	4,960 (40%)
	25–29	2,771 (22%)	2,867 (21%)	2,585 (21%)
	30–34	2,137 (17%)	2,278 (17%)	2,058 (17%)
	35–39	1,453 (12%)	1,705 (13%)	1,535 (13%)
	40–44	1,175 (9%)	1,295 (10%)	1,096 (9%)
HIV-status^†^	Positive	2,071 (18%)	2,215 (18%)	2,099 (18%)
	Negative	9,745 (82%)	10,320 (82%)	9,372 (82%)

All percentages rounded to the nearest whole number, where possible.

^¶^Denominator for all column percentages shown in the column (unless otherwise indicated).

*Denominator is the total number contributing person time each year (row percentage).

^×^Geometric mean of estimated community HIV prevalence among the population aged 18–44 years using HIV prevalence estimates at PC0 which were standardised using the population structure of the communities.

^Ø^Geometric mean of estimated minimum population size, based on census conducted by the study team in 2013.

^‡^Age in years at PC0.

^†^Measured in the study cohort with HIV-status based on laboratory HIV-testing.

PC, population cohort; SA, South Africa; ND, not determined.

**Fig 2:**
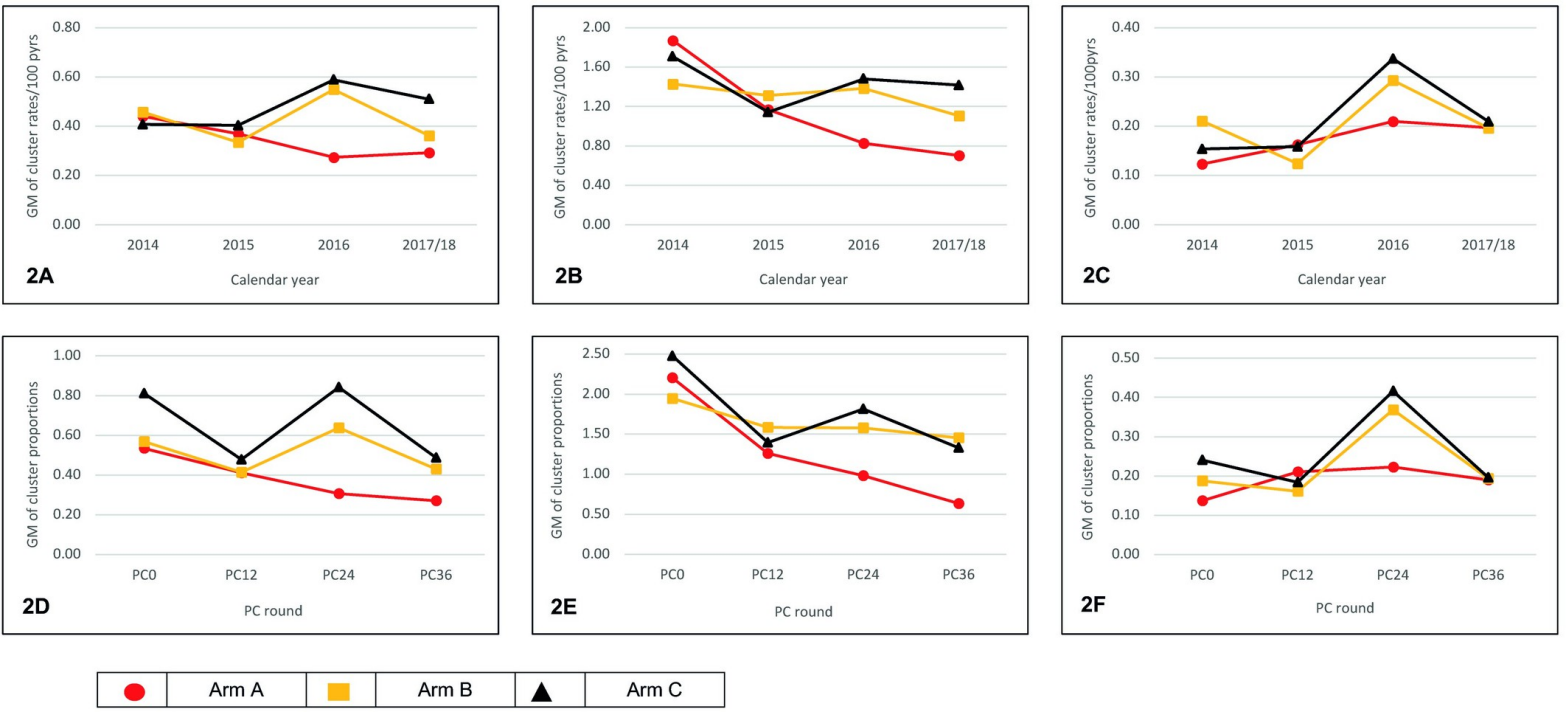
Geometric mean of the cluster rates (2A to 2C) of self-reported TB treatment by year and study arm in the cohort analysis, and, geometric mean of the cluster proportions (2D to 2F) of self-reported TB treatment by PC visit and study arm in the cross-sectional analysis, among PC participants from all 21 HPTN 071 (PopART) communities. GM, geometric mean; pyrs, person years; PC, population cohort; 2A, total population; 2B, people living with HIV; 2C, people who were HIV negative; 2D, total population; 2E, people living with HIV; 2F, people who were HIV negative.

The overall incidence of self-reported TB was 0.53/100 person-years (154 self-reported TB/28,847 person-years) in 2014; 0.46/100 person-years (112/24,151) in 2015, 0.64/100 person-years (136/21,193) in 2016, and 0.47/100 person-years (92/19,544) in 2017/2018. While self-reported TB incidence (geometric mean across communities) showed year-on-year fluctuations, there were some discernible patterns (Fig 2 and S11 Appendix and Table 3). Incidence was similar across study arms in 2014 and 2015 (Table 4). Over time, incidence in arm C increased, from 0.41 in 2014 to 0.59 and 0.51/100 person-years in 2016 and 2017/2018, respectively. In arm A, incidence decreased from 0.44 in 2014 to 0.27 and 0.29/100 person-years in 2016 and 2017/2018, respectively; the adjusted RR compared with arm C was 0.48 (95% CI 0.28 to 0.81; *p* = 0.01) in 2016 and 0.58 (95% CI 0.27 to 1.22; *p* = 0.13) in 2017/2018. In arm B incidence varied, ranging between 0.33 and 0.55/100 person-years; incidence in arms B and C was similar at all time points.

**Table 3:** The rate of self-reported TB treatment by study arm and calendar year in the cohort analysis from all 21 HPTN 071 (PopART) communities.

	Total population rate* (d/pyrs)			PLHIV rate* (d/pyrs)			HIV negative rate* (d/pyrs)		
Self-reported TB	Arm A	Arm B	Arm C	Arm A	Arm B	Arm C	Arm A	Arm B	Arm C
2014	0.44 (45/9,473)	0.46 (56/9,985)	0.41 (53/9,389)	1.87 (26/1,397)	1.43 (22/1,526)	1.71 (29/1,463)	0.12 (12/7,392)	0.21 (20/7,755)	0.15 (15/7,202)
2015	0.37 (40/7,865)	0.33 (33/8,161)	0.40 (39/8,124)	1.17 (24/1,590)	1.31 (23/1,624)	1.14 (22/1,764)	0.16 (14/5,894)	0.12 (9/6,208)	0.16 (15/5,921)
2016	0.27 (31/6,827)	0.55 (49/7,643)	0.59 (56/6,722)	0.83 (17/1,453)	1.38 (27/1,554)	1.48 (32/1,521)	0.21 (13/5,093)	0.29 (20/5,820)	0.34 (22/4,878)
2017/2018	0.29 (23/6,305)	0.36 (34/7,064)	0.51 (35/6,175)	0.70 (11/1,458)	1.11 (20/1,552)	1.42 (20/1,483)	0.20 (12/4,684)	0.20 (13/5,371)	0.21 (15/4,567)

*Rate calculated as the geometric mean of the cluster rates in each arm expressed per hundred person years.

TB, tuberculosis; PLHIV, people living with HIV; d, total number self-reporting TB treatment; pyrs, person years; PC, population cohort; d/pyrs, total number self-reporting TB treatment/total number of person years contributed by PC-participants during each calendar year (2014, 2015, 2016, and 2017/2018) in each study arm.

**Table 4:** The effect of the HPTN 071 (PopART) intervention on the incidence rate of self-reported TB treatment, by calendar year among PC participants.

		2014			2015			2016			2017/2018		
Population	Adjusted for	RR	95% CI	*p*-Value	RR	95% CI	*p*-Value	RR	95% CI	*p-*Value	RR	95% CI	*p*-Value
**Arm A vs. C**													
Total population	Triplet	1.08	(0.51–2.30)	0.83	0.91	(0.36–2.32)	0.83	0.47	(0.28–0.78)	0.007	0.57	(0.28–1.14)	0.10
	Triplet and HIV	1.10	(0.52–2.33)	0.78	0.97	(0.37–2.59)	0.95	0.48	(0.28–0.81)	0.01	0.58	(0.27–1.22)	0.13
PLHIV	Triplet	1.10	(0.49–2.47)	0.81	1.02	(0.52–2.01)	0.94	0.56	(0.29–1.08)	0.08	0.50	(0.26–0.95)	0.04
PLHIV*	Triplet	1.13	(0.52–2.46)	0.74	1.03	(0.50–2.14)	0.92	0.55	(0.28–1.05)	0.06	0.50	(0.26–0.95)	0.04
**Arm B vs. C**													
Total population	Triplet	1.13	(0.53–2.42)	0.72	0.84	(0.33–2.14)	0.68	0.93	(0.56–1.55)	0.77	0.71	(0.35–1.42)	0.30
	Triplet and HIV	1.11	(0.53–2.35)	0.76	0.95	(0.36–2.54)	0.92	0.97	(0.57–1.64)	0.90	0.72	(0.34–1.52)	0.35
PLHIV	Triplet	0.84	(0.37–1.89)	0.64	1.15	(0.59–2.26)	0.66	0.93	(0.48–1.80)	0.82	0.78	(0.41–1.50)	0.43
PLHIV*	Triplet	0.95	(0.44–2.07)	0.89	1.25	(0.60–2.59)	0.52	0.92	(0.48–1.77)	0.79	0.78	(0.41–1.50)	0.43

*HIV-status based on the sensitivity analysis: If HIV–positive for a calendar year and HIV-status was not determined in the preceding year, HIV-status in the preceding year assumed to be positive.

TB, tuberculosis; RR, rate ratio; 95% CI, 95% confidence interval; PLHIV, people living with HIV.

Among PLHIV, overall self-reported TB incidence was 1.76/100 person-years (77 self-reported TB/4,385 person-years) in 2014, 1.39/100 person-years (69/4,977) in 2015, 1.68/100 person-years (76/4,528) in 2016, and 1.14/100 person-years (51/4,493) in 2017/2018. In 2014 and 2015, incidence in arms C and A was similar (Fig 2 and S11 Appendix and Tables 3 and 4). In arm C, incidence decreased gradually from 1.71/100 person-years in 2014 to 1.48 and 1.42/100 person-years in 2016 and 2017/2018, respectively. In arm A, decreases in incidence were large and sustained, from 1.87/100 person-years in 2014 to 0.83/100 person-years in 2016, and 0.70/100 person-years in 2017/2018; the RR compared to arm C was 0.56 (95% CI 0.29 to 1.08; *p* = 0.08) in 2016 and 0.50 (95% CI 0.26 to 0.95; *p* = 0.04) in 2017/2018. In arm B, incidence decreased slightly from 1.43/100 person-years in 2014 to 1.38/100 person-years in 2016. Incidence in arms B and C was similar over this period. In 2017/2018, incidence in arm B fell to 1.11/100 person-years, showing separation from arm C; the RR compared to arm C was 0.78 (95% CI 0.41 to 1.50; *p* = 0.43). Sensitivity analysis, changing the HIV–positive case definition, did not alter findings. Among those HIV–negative (S11 Appendix), the number of events was very low, with null events in multiple communities, over multiple calendar years; self-reported TB incidence varied over time across study arms.

### Cross-sectional analysis

All 686 events were used to determine the proportion self-reporting TB at each PC-visit. In arms C and B, the overall proportion (geometric mean across communities) followed a similar variable pattern (Fig 2 and S12 Appendix and Tables 5 and 6). In arm A, the proportion self-reporting TB decreased steadily at each PC-visit. The adjusted PR compared with arm C was 0.44 (95% CI 0.23 to 0.85; *p* = 0.02) at PC24 and 0.58 (95% CI 0.30 to 1.10; *p* = 0.09) at PC36. The estimated coefficient of between-community variation k was in the range of approximately 0.0 to 0.20 between PC12 and PC36, after accounting for between-arm and between-triplet variation (S13 Appendix).

**Table 5:** The proportion self-reporting TB treatment by study arm and PC visit in the cross-sectional analysis from all 21 HPTN 071 (PopART) communities.

	Total population %† (d/N)			PLHIV %† (d/N)			HIV negative %† (d/N)		
Self-reported TB	Arm A	Arm B	Arm C	Arm A	Arm B	Arm C	Arm A	Arm B	Arm C
PC0	0.53 (77/12,671)	0.57 (82/13,404)	0.81 (120/12,399)	2.20 (55/2,583)	1.95 (56/2,734)	2.48 (81/2,687)	0.14 (20/9,594)	0.19 (22/10,235)	0.24 (37/9,301)
PC12	0.41 (48/8,234)	0.41 (45/8,572)	0.48 (49/8,484)	1.26 (27/1,661)	1.58 (31/1,660)	1.40 (26/1,765)	0.21 (15/5,781)	0.16 (12/6,210)	0.18 (16/5,678)
PC24	0.31 (37/6,938)	0.64 (55/7,873)	0.84 (68/6,867)	0.98 (21/1,459)	1.58 (26/1,608)	1.82 (28/1,512)	0.22 (15/4,931)	0.37 (24/5,691)	0.42 (28/4,672)
PC36	0.27 (22/6,623)	0.43 (46/7,416)	0.49 (37/6,383)	0.63 (10/1,549)	1.45 (28/1,637)	1.33 (22/1,572)	0.19 (12/4,873)	0.19 (16/5,587)	0.20 (15/4,651)

^†^Proportion calculated as the geometric mean of the cluster proportions in each arm.

TB, tuberculosis; PLHIV, people living with HIV; %, proportion; d, total number self-reporting TB treatment; *N*, total number seen at each PC-visit; PC, population cohort; d/N, total number self-reporting TB treatment/total number of PC-participants seen at each PC-visit (PC0, PC12, PC24, and PC36) in each study arm.

**Table 6:** The effect of the HPTN 071 (PopART) intervention on the proportion self-reporting TB treatment, by PC visit, among PC participants.

		PC0			PC12			PC24			PC36		
Population	Adjusted for	PR	95% CI	*p*-Value	PR	95% CI	*p*-Value	PR	95% CI	*p*-Value	PR	95% CI	*p*-Value
**Arm A vs. C**													
Total population	Triplet	0.66	(0.37–1.19)	0.15	0.87	(0.40–1.87)	0.70	0.36	(0.20–0.64)	0.002	0.56	(0.31–1.02)	0.06
	Triplet and HIV	0.68	(0.38–1.20)	0.17	0.92	(0.41–2.07)	0.83	0.44	(0.23–0.85)	0.02	0.58	(0.30–1.10)	0.09
PLHIV	Triplet	0.89	(0.50–1.58)	0.66	0.90	(0.47–1.72)	0.73	0.54	(0.30–0.99)	0.05	0.48	(0.23–0.99)	0.05
**Arm B vs. C**													
Total population	Triplet	0.69	(0.38–1.25)	0.20	0.87	(0.40–1.87)	0.69	0.76	(0.42–1.35)	0.31	0.89	(0.49–1.63)	0.69
	Triplet and HIV	0.67	(0.38–1.18)	0.15	0.99	(0.44–2.21)	0.97	0.87	(0.45–1.69)	0.66	0.91	(0.48–1.73)	0.76
PLHIV	Triplet	0.78	(0.44–1.40)	0.38	1.13	(0.59–2.16)	0.68	0.87	(0.48–1.58)	0.62	1.09	(0.53–2.26)	0.80

TB, tuberculosis; PC, population cohort; PR, prevalence ratio; 95% CI, 95% confidence interval; PLHIV, people living with HIV.

Among PLHIV, the proportion self-reporting TB in arms A and B was similar to arm C at PC0 and PC12 (Fig 2 and S12 Appendix and Tables 5 and 6). Between PC12 and PC36, the proportions in arm A decreased steadily. The PR compared with arm C was 0.54 (95% CI 0.30 to 0.99; *p* = 0.05) at PC24 and 0.48 (95% CI 0.23 to 0.99; *p* = 0.05) at PC36. In arm B, while the proportions gradually decreased between PC12 and PC36, the proportions in arms B and C were similar at these PC-visits. Among those HIV–negative (S12 Appendix), the number of events at PC-visits was very low with null events in multiple communities. Self-reported TB incidence varied over time across study arms.

## Discussion

In this preplanned analysis of a large cluster-randomised trial in sub-Saharan Africa, compared to standard-of-care, we found a decrease in self-reported TB incidence among PLHIV following the roll-out of community-wide UTT and systematic TB screening in arm A, which received the full intervention package from the start. There was also some evidence that this translated to a decrease in self-reported TB incidence overall in the population, although confidence intervals around some effect estimates with less follow-up time/lower sample sizes were wide and crossed 1. There were insufficient events to determine if the intervention had an effect on self-reported TB incidence among those HIV–negative.

With TB screening, we anticipated large initial increases in self-reported TB in the intervention arms [28], which we did not see. Decreases in self-reported TB incidence after the first intervention round among PLHIV in arm A suggests UTT was the main driver of the intervention effect. Our findings were in keeping with mathematical modelling predictions of the impact of UTT on HIV-associated TB incidence [21].

To date, 4 large HIV treatment as prevention trials have been conducted [30]. It is unlikely that trials of their scale and scope will ever be conducted again. Of these, only 1 trial other than HPTN 071 (PopART), the Sustainable East Africa Research in Community Health (SEARCH) trial, evaluated the impact of UTT on TB [31]. However, this was a post hoc analysis, which therefore requires cautious interpretation. Nonetheless, the TB notification rate ratio in the intervention (UTT) compared to the control arm among PLHIV was 0.41 (95% CI 0.19 to 0.86); there was no effect among those HIV–negative. Our results confirm these preliminary findings and support the role of UTT in TB control in sub-Saharan Africa.

Self-reported TB should reflect TB notifications, which for small geographic areas typically show year-on-year fluctuations as seen with data from the standard-of-care (arm C) communities [32]. These fluctuations were mainly among HIV–negative individuals (due to the small number of events). Among PLHIV, there were discernible trends across all arms, with limited fluctuation, and results consistent between the cohort and cross-sectional analysis, lending weight to the robustness of the findings. Self-reported treatment was used as the outcome, rather than “told they had TB” (i.e., potential diagnoses), as the questionnaire was designed to determine treatment starts. The outcome was based on self-report [18,33–36]. Research staff were extensively trained and supervised, with in-built prompts and skip patterns in electronic data capture likely to limit errors in questioning and documenting responses. Misclassification through underreporting due to stigma or social-desirability bias was possible but would be expected to be similar across study arms [37,38]. In a cohort study, this should not bias the RR, with the ratio representing the intervention effect on underlying TB incidence. If the intervention changed TB-stigma, the direction of the effect given the community-engagement and participatory nature of the trial would likely reduce stigma and therefore under-reporting in intervention communities. Self-reported TB in standard-of-care communities would be lower, as a proportion of true treatment starts, with impact underestimated. Treatment for other conditions being erroneously reported as TB [37,38] was unlikely because TB knowledge was common across communities, data collection was structured, with information sharing during the process through in-built prompts and repeated in the same closed cohort over time, and information on TB treatment (which takes 6 to 8 months) was only collected for the 12 months preceding a PC-visit. Misclassification of TB preventive therapy (TPT) use as TB treatment was also unlikely as PC-participants were specifically asked about TPT use at each PC-visit, research staffs were trained on how to administer the TB treatment versus TPT questions, and TPT use by routine services was suboptimal during the study period. Any possible over-reporting would also be expected to be similar across study arms biasing the RR in a cohort study towards the null.

Using self-reported TB in PC as the outcome in our study had some strengths. Measuring the impact of interventions on TB incidence is usually not feasible, but this is the critical outcome for drawing causal inferences about TB control interventions. In well-functioning health systems, where nearly all people with TB are diagnosed, treated, and events captured through quality-assured routine surveillance systems, TB notifications can be used as a proxy for TB incidence [39]. But this is not the case in sub-Saharan Africa and the availability and quality of TB notification data varied substantially across study community health centres. Further, people with TB living in study communities often started TB treatment outside community health centres, and therefore using health centre data would have underestimated TB notifications. These care-seeking behaviours also varied by community. When using self-reported TB in the PC, while some under-reporting was possible, the estimated impact should, nonetheless, reflect the minimum impact of the intervention on underlying TB incidence.

When national guidelines for ART initiation recommended a CD4 cell threshold of <500 cells/μl, the self-reported TB incidence among PLHIV in intervention arm B (where ART start followed guidelines) and the standard-of-care arm was similar. While we do not have CD4 data for PC-participants, the proportion of PLHIV with viral suppression was higher in arm A than B, and in both intervention arms than the standard-of-care arm [24]. After national guidelines changed to universal ART in 2016, self-reported TB incidence in arm B showed a nonsignificant decrease compared to the standard-of-care arm. But there was insufficient follow-up to determine if effects were sustained. Nonetheless this, together with findings from arm A, suggests that universal HIV testing alongside universal ART was critical to achieving intervention benefits quickly. Going forward, identifying models of universal HIV testing and linkage-to-care that are acceptable, cost-effective, and reflect the local TB/HIV epidemiology will be important, if countries want to translate trial findings to local benefits.

Despite large decreases in self-reported TB incidence in arm A compared to standard-of-care communities, absolute incidence in arm A remained high (geometric means approximately 300/100,000 overall and approximately 800/100,000 among PLHIV). The trial duration was short, and therefore we were unable to determine the longer-term impact of sustained UTT. Mathematical modelling predicts that following an initial steep drop in HIV-associated TB incidence with UTT, incidence will subsequently fall more slowly [21]. This is because PLHIV on ART live longer [2]. While ART decreases their risk of incident TB, it does not return it to that of HIV–negative individuals, giving a relatively high cumulative lifetime risk of TB [2]. This is coupled with the background risk of TB among those HIV–negative, who contribute >30% of all incident TB in sub-Saharan Africa [1]. Therefore, scale-up of other TB prevention interventions, such as TPT in risk groups (e.g., PLHIV) as recommended by WHO, is needed to prevent TB at the individual-level, which may also translate to population-level benefits [40]. While systematic TB screening is recommended by WHO in high TB prevalence settings [22], we found no evidence that this increased the proportion of individuals who reported starting TB treatment. Possible explanations include the low sensitivity of symptom screening for prevalent TB [22], and the use of sputum smear in the diagnostic algorithm, which has lower sensitivity than other diagnostic methods [41]. Screening with chest-radiographs, and the routine widespread use of GeneXpert MTB/RIF for TB diagnosis may overcome some of these limitations [22,41].

Limitations of our study include limited covariate adjustment due to the small number of events and high losses to follow-up over time. While residual confounding and selection bias cannot be excluded, the characteristics of individuals seen, and proportions seen at each calendar year/PC-visit did not differ by study arm. The cohort analysis used longitudinal data on self-reported TB and treatment start dates, allowing incidence rates to be estimated. However, it may have been biased by errors in reported dates and gaps in follow-up between PC-visits where outcome status was unknown. But conclusions from the cross-sectional analysis (based on fewer assumptions and done to check the robustness of the cohort analysis findings) were very similar, supporting the overall findings. HIV-status was determined at each PC-visit and not at TB treatment start; therefore, some misclassification was likely. However, findings were similar using different approaches to classifying HIV-status and so the effect of any misclassification was likely to be small. HIV-status was defined using all available HIV data (prevalent and incident) to capture the full effect of the interventions on self-reported TB incidence among PLHIV. However, because UTT was shown to decrease HIV incidence, this may have decreased the comparability between PLHIV across study arms. But the degree of any bias was likely to be very small because HIV incidence was very low (approximately 1.4 per 100 person years) compared with prevalence (approximately 18%) and therefore, the number of people with incident HIV at follow-up was very small compared with those who were HIV positive at baseline. Further the intervention effect on HIV incidence in arm A compared to arm C was very modest (7% reduction in HIV incidence), and the characteristics of PLHIV at each calendar year/PC-visit did not differ by study arm. PC-participants were aged 18 to 44 years at enrolment; therefore, findings cannot be generalised to the population as a whole.

In conclusion, in this cluster-randomised trial in sub-Saharan Africa, compared to standard-of-care, we found a decrease in self-reported TB incidence among PLHIV following the roll-out of community-wide UTT and systematic TB screening in arm A, which received the full intervention package from the start. There was also some evidence that this translated to a decrease in self-reported TB incidence overall in the population. UTT could contribute to controlling TB in addition to HIV in high TB/HIV burden settings.

## Supporting information

S1 Appendix Figure: The 3 study arms. (DOCX)

S2 Appendix Figure: The PopART HIV/TB intervention delivered at each intervention round, over 3 rounds between November 2013 and December 2017 in study arms A and B. (DOCX)

S3 Appendix Figure: HPTN 071 (PopART) study timelines showing intervention rounds, intervention components, ART eligibility criteria, population cohort rounds (PC0-PC36), and the observation period for the analysis in this TB study for each population cohort visit (generated as 14 months before the date of the PC-visit). (DOCX)

S4 Appendix Statistical considerations detailing the predefined proposed analyses, outcomes, power calculations, and analysis plan. (DOCX)

S5 Appendix Table: Characteristics of population cohort participants enrolled at PC0 and follow-up at PC12, PC24, and PC36, respectively, from all 21 HPTN 071 (PopART) communities: overall and by study arm. (DOCX)

S6 Appendix Figure: Consort flow diagram showing the PC participants from all 21 HPTN 071 (PopART) communities that contributed to the cross-sectional analysis. (DOCX)

S7 Appendix Table: Characteristics of individuals not seen and seen (at least once during follow up and at each follow up PC visit [PC12, PC24, AND PC36, respectively]). (DOCX)

S8 Appendix Table: Characteristics of population cohort participants from all 21 HPTN 071 (PopART) communities, who were HIV–positive (based on laboratory HIV-testing) and contributed to the cross-sectional and cohort analysis: overall and by study arm. (DOCX)

S9 Appendix Table: Number and proportion self-reporting being told they had TB and starting TB treatment with duration between visit date and MM/YYYY of TB treatment start ≤14 months, by population cohort visit, in the cohort enrolled at PC0 (*N* = 38,474) from all 21 HPTN 071 (PopART) communities. (DOCX)

S10 Appendix Table: Characteristics of population cohort participants contributing person time to the cohort analysis from all 21 HPTN 071 (PopART) communities, by calendar year and study arm. (DOCX)

S11 Appendix Table: Incidence rate of self-reported TB treatment, by community, triplet, study arm and calendar year (2014 to 2017/2018) among population cohort participants from all 21 HPTN 071 (PopART) communities. (DOCX)

S12 Appendix Table: Proportion self-reporting TB treatment, by community, triplet, study arm, and population cohort visit among population cohort participants from all 21 HPTN 071 (PopART) communities. (DOCX)

S13 Appendix. Table The estimated coefficient of between-community variation k at each population cohort visit (PC0, PC12, PC24, and PC36, respectively). (DOCX)

S1 CONSORT Checklist CONSORT 2010 checklist of information to include when reporting a randomised trial, with extension to cluster randomised trials checklist items (shown in italics) taken from BMJ 2012;345:e5661. (DOCX)

## Abbreviations

aRR: adjusted rate ratio
ART: antiretroviral therapy
CI: confidence interval
PC: population cohort
PLHIV: people living with HIV
PR: prevalence ratio
RR: rate ratio
TB: tuberculosis
TPT: TB preventive therapy
UTT: universal testing and treatment
WHO: World Health Organization
