# Supplementary material for: Incidence of self-reported tuberculosis treatment with community-wide universal testing and treatment for HIV and tuberculosis screening in Zambia and South Africa: A planned analysis of the HPTN 071 (PopART) cluster-randomised trial
Source: PLoS Med. 2024 May 31;21(5):e1004393. doi: 10.1371/journal.pmed.1004393 (PMC11142425; doi:10.1371/journal.pmed.1004393)
Supplement: S1 Appendix — (DOCX) [file pmed.1004393.s001.docx]

**S1 Appendix**

**Universal testing for HIV**

**+ ART according to national guidelines**

**UTT 2016**

**TB screening**

**ART according to national guidelines**

**→**

**Universal treatment for HIV in 2016**

**21 communities in total; 7 per study arm.**

**Population cohort: ~2500 random sample of adults aged 18-44 years from each community**

**UTT for HIV**

**TB screening**

**Full PopART intervention**

**PopART intervention**

**Standard of care**

**4 Zambian triplets**

**3 South African triplets**

**Figure: The three study arms**

UTT=universal testing and treatment for HIV; ART=antiretroviral therapy.

There were 2 intervention arms – arm A (7 communities) and B (7 communities). Arm A received the full intervention package, which included universal testing for HIV, with universal treatment for HIV (irrespective of CD4 cell count) from 2013 and community-wide TB screening from 2013. In arm B there was universal testing for HIV, but ART start was according to national guidelines, which changed to universal treatment in April 2016 in Zambia and October 2016 in South Africa. There was community-wide TB screening in arm B from 2013. Therefore from April 2016 in Zambia and October 2016 in South Africa, the arm A and B communities were the same; giving a full intervention year – 2017 – in which there was no difference in the intervention package delivered in the 2 intervention arms. Arm C (7 communities), the control received the standard of care through routine services. ART initiation criteria followed national guidelines, changing to universal treatment in 2016. There was no universal testing for HIV or TB screening in arm C communities.
