# Supplementary material for: Incidence of self-reported tuberculosis treatment with community-wide universal testing and treatment for HIV and tuberculosis screening in Zambia and South Africa: A planned analysis of the HPTN 071 (PopART) cluster-randomised trial
Source: PLoS Med. 2024 May 31;21(5):e1004393. doi: 10.1371/journal.pmed.1004393 (PMC11142425; doi:10.1371/journal.pmed.1004393)
Supplement: S2 Appendix — (DOCX) [file pmed.1004393.s002.docx]

**S2 Appendix**

House-to-house visits

Community HIV-care providers

**x3 community-wide intervention rounds between 11/2013 and 12/2017 in arms A and B**

**round 1: Nov-2013 to Jun-2015**

**round 2: Jul-2015 to Sept-2016**

**round 3: Oct-2016 to Dec-2017**


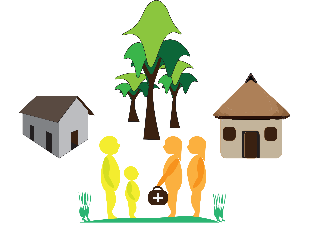

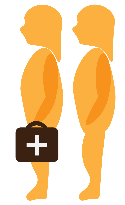


| 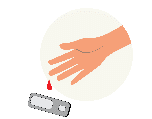  **HIV** | ****  **TB** |
| --- | --- |
| **HIV-testing at each intervention round using rapid tests (universal testing)**  **Referred to community health facility if HIV-positive**   - **Arm A – immediate ART start (universal treatment)** - **Arm B – ART start according to national guidelines. Became immediate ART (universal treatment) in 2016.** | **TB symptom screening at each intervention round using a questionnaire**   - **Cough ≥2 weeks *or*** - **Night sweats *or*** - **Unintentional weight loss ≥1.5Kg in <1 month**   **If symptomatic sputum collected:**  ***Zambia intervention round 1-3***   - **Smear if HIV negative** - **Xpert MTB/RIF if HIV positive/status unknown**   ***South Africa intervention round 1***   - **Smear if HIV negative** - **Xpert MTB/RIF if HIV positive/status unknown**   ***South Africa intervention round 2-3***   - **Xpert MTB/RIF irrespective of HIV-status**   **Referred to community health facility for TB treatment if sputum positive for TB** |

**Figure: The PopART HIV/TB intervention delivered at each intervention round, over 3 rounds between November 2013 and December 2017 in study arms A and B**

Trained community health workers called Community HIV-Care Providers delivered the house-to-house community-wide intervention. Each household in the community was visited at least 3 times over the intervention period. In addition to screening and referral activities, the Community HIV-Care Providers followed-up on all referrals and treatment adherence support was also provided.
