# Supplementary material for: Incidence of self-reported tuberculosis treatment with community-wide universal testing and treatment for HIV and tuberculosis screening in Zambia and South Africa: A planned analysis of the HPTN 071 (PopART) cluster-randomised trial
Source: PLoS Med. 2024 May 31;21(5):e1004393. doi: 10.1371/journal.pmed.1004393 (PMC11142425; doi:10.1371/journal.pmed.1004393)
Supplement: S4 Appendix — (DOCX) [file pmed.1004393.s004.docx]

**S4 Appendix**

**Statistical considerations detailing the pre-defined proposed analyses, outcomes, power calculations, and analysis plan.**

Rationale: self-reported TB treatment (self-reported TB) among population cohort (PC) participants should reflect TB notifications. Therefore, comparing self-reported TB in intervention arms compared to the control, should reflect the effect of the HPTN 071 (PopART) intervention on **all** TB notifications (bacteriologically confirmed and clinically diagnosed, pulmonary and extrapulmonary TB). Linkage of self-reported TB to routine TB notification data would have allowed treatment starts to be verified and the effect of the HPTN 071 (PopART) intervention on **bacteriologically confirmed** TB notifications to be explored.

TB outcomes among PC participants which were pre-defined and documented for this work:

1) Primary outcome: Notified bacteriologically confirmed (smear, Xpert and/or culture positive) pulmonary TB incidence. Bacteriologically confirmed TB was to be determined through linkage of PC data to routine TB notification data.

2) Secondary outcome: All (bacteriologically confirmed and clinically diagnosed, pulmonary and extrapulmonary) notified TB incidence. All TB was to be determined through self-reported TB and by using self-reported TB linked to TB notification data.

3) Time period for comparison: By year and overall. The overall analysis was restricted to the last 24 months of follow-up (2017-2018). This was done to exclude large initial rises in TB notifications expected with TB screening. Intervention effects were likely to accrue over time with repeated rounds of Universal Testing and Treatment for HIV (UTT) and TB screening and therefore the effect was likely to be maximal (as evidenced through preliminary mathematical modelling work undertaken for HPTN 071) in the last intervention years.

Mathematical modelling to inform study power calculations: The complexity of the multiple components of the intervention made it difficult to estimate its population impact on notified TB incidence. To determine the likely effect of the HPTN 071 (PopART) interventions on TB epidemiology, a deterministic, compartmental mathematical model was developed in R. Run over one-month time-steps, it captured the intervention and control arms and allowed comparisons across arms over time.

In the intervention arms, the model captured the effects of 3 rounds of UTT and TB screening over 4 years on TB epidemiology. Baseline data for the communities (e.g. TB disease prevalence and annual risk of TB infection) were available from the ZAMSTAR trial[1], conducted in the same study areas between 2006-2010. This allowed TB disease incidence at baseline to be estimated (incidence=prevalence/duration). From this equilibrium at baseline, the model was run, capturing the dynamic monthly and cumulative changes in notified bacteriologically confirmed pulmonary TB incidence in study arms and relative to one another.

The model predicted a ~40-55% decrease in notified bacteriologically confirmed pulmonary TB incidence in the intervention arms over the last 24 months if annual TB screening rounds identified 20-30% of previously undiagnosed TB cases and 40-60% of untreated people living with HIV (PLHIV) were identified and linked to care. An average notified bacteriologically confirmed pulmonary TB incidence rate of 0.87 per 100-person years in the control arm was also predicted.

The coefficient of between-community variation k was assumed to be in the range 0.20-0.25[1]. In the Zamstar trial, within each country (Zambia and South Africa) trial communities were grouped into 2 strata based on estimates of TB infection among schoolchildren at the start of the trial. Thus, there were 4 strata (2 countries, and 2 strata within each country). In the TB prevalence survey conducted in 2010 to measure the primary endpoint of the Zamstar trial, the coefficient of between-community variation in TB prevalence – among communities in the same strata - was estimated to be k=0.29. Taking account of key covariates, however, the data were consistent with a lower value of k in the range 0.20-0.25. In HPTN 071 (PopART), communities were pair-matched on geographical area and adult HIV prevalence (a stronger risk factor for TB incidence than TB prevalence). Further, the analysis planned to adjust for TB risk factors, further reducing between-community variation.

Study power was calculated using standard formulae for pair-matched cluster-randomised trials. The reduction in bacteriologically confirmed pulmonary TB incidence in the intervention arms was assumed to be in the range of 40-50%. The average notified bacteriologically confirmed pulmonary TB incidence rate was assumed to be 0.87 per 100-person years in the control arm. The average estimated person years of follow-up in each community of the Population Cohort was estimated to be ~1964-person years (estimated for the parent HPTN071 [PopART] trial), over the last 24 months of follow-up. With k=0.2, this gave a power of 76-85% to detect a 45-50% decrease in notified bacteriologically confirmed pulmonary TB incidence in the intervention arm. The power was 68-78% when k=0.25.

Analytic approach: All data preparation and analysis were undertaken in Stata. To analyse TB data within PC, the start of the HPTN 071 (PopART) intervention was defined as January 1, 2014. The aim was to include all PC participants in the analysis (including those newly enrolled at PC12N and PC24N).

The data were to be analysed in 2 ways – cohort and cross sectional. The cohort analysis allowed full use of the data – in longitudinal format. Incidence rates of TB could be estimated. This was the primary analysis. The cross-sectional approach analysed each PC visit as an independent cross-sectional sample. The proportion with TB was estimated for each PC visit. This was the secondary analysis.

Statistical inference used the 2-stage approach recommended for cluster-randomized trials with <15 clusters/arm[2,3] – see further details on the 2-stage approach below. The aim was to include triplet, HIV status, age, and sex (with an interaction term between age and sex) as covariates at the first stage of the analysis. Formal cross arm comparisons would be Arm A versus C and Arm B versus C, separately. Data were to be analysed overall and by HIV status (for PLHIV and those who are HIV negative separately).

Changes to original research plans:

March 2022: There were challenges with linking PC data to TB notification data in South Africa and Zambia due to the quality of TB notification data available for use. In South Africa, there were shortfalls in TB notifications captured through the Electronic TB Registers, across multiple communities and multiple calendar years, during the study period. In Zambia all TB notification data were in paper form and had to be captured electronically. There were missing registers for parts of/whole calendar years for multiple communities. Therefore, in both South Africa and Zambia, PC data could not be linked to TB notification data, for large periods of PC follow-up. The analysis was therefore restricted to self-reported TB alone. Details of the analysis plan for self-reported TB are presented.

March 2022: Community HIV-care Providers’ intervention data were summarised during the trial. This showed significant churn within the communities (representing ~1/3 of the total population in intervention communities in the 3^rd^ intervention round, during which data on migration pattern were collection. These figures were likely to be generalisable to the previous intervention rounds and to control communities). Therefore, the TB analysis was restricted to PC participants enrolled at PC0 only. This was because TB incidence in PC participants enrolled at later visits (PC12N and PC24N) may not have been representative of study community incidence as:

1. In intervention communities, the migration pattern could have represented movement from areas not receiving the intervention, to intervention areas.
2. How long a PC participant had resided within the community was not an eligibility criterion for enrolment and data on migration was not captured in the PC questionnaire.
3. The questionnaire asked about TB treatment in the 12 months before a PC visit. As TB disease takes months/years to develop following infection, the reported TB treatment start could be for a transmission event that may not represent transmission occurring in the study communities.

August 2022: There were insufficient self-reported TB events among those who were HIV negative. There were no events among HIV negative individuals in six communities in 2014, eight communities in 2015, two communities in 2016 and five communities in 2017/18. Therefore, rates were summarised, but formal cross-arm comparisons were not conducted. Due to the small number of events during later calendar years (especially in 2017/18 in arm A), the total population analysis only included triplet and HIV status as covariates (without adjusting for age and sex). Analyses among PLHIV included triplet alone.

Outcome definition: To determine self-reported TB the variables (listed) in the TB screening section of the PC questionnaire were used. All these variables were linked through skip patterns.

| TBTOLD | In the last 12 months, have you been told that you have TB? |
| --- | --- |
| TBTRT | PC0: Have you started TB treatment?  PC12-36: Have you started TB treatment in the last 12 months? |
| TBTRTMM | When did you start TB treatment? Please give the month and year. |
| TBTRTYY | When did you start TB treatment? Please give the month and year. |

Variables listed in the TB screening section of the PC questionnaire which were not used:

| TBASK | During the past 12 months, has a health worker, at the clinic or in the community, asked you questions about TB such as whether you have a cough, fever, night sweats, weight loss?  *About TB screening. Not linked to TB treatment related questions.* |
| --- | --- |
| TBTXT | Do you have your TB number?  *TB patients would only have their TB number if: given a TB card, TB number was documented on the card (not always done), they were still on TB treatment (card usually taken back at the end of TB treatment) and the TB card was not lost. Therefore, not having a number did not mean individual were not on or had not been on TB treatment.* |
| TBNUM | Do you have your TB number?  *As above* |
| TBIPT | Have you ever/are you currently taking isoniazid preventive treatment to prevent TB?  *Not relevant for determining TB treatment start. Not linked to TB treatment related questions* |

MM/YYYY documented?

PC0 MM/YYYY: missing; >14 months between self-reported treatment start MM/YYYY and PC visit date

PC12-36 MM/YYYY: >14 months between self-reported treatment start MM/YYYY and PC visit date

PC0: ≤14 months between self-reported treatment start MM/YYYY and PC-visit date

PC 12-36: Missing MM/YYYY *OR* ≤14 months between self-reported treatment start MM/YYYY and PC visit date

**SELF-REPORTED TB**

In the last 12 months, have you been told that you have TB?

Response options: Yes; No; Don’t know; No answer

No; Don’t know; No answer:

**STOP HERE AND SKIP TO NEXT SECTION IN QUESTIONNAIRE**

PC0: Have you started TB treatment?

PC 12-36: Have you started TB treatment in the last 12 months?

Response options: Yes; No; Don’t know; No answer

Yes

No; Don’t know; No answer:

**STOP HERE AND SKIP TO NEXT SECTION IN QUESTIONNAIRE**

Yes

When did you start TB treatment?

Please give the month and year

Figure 3: Flow of questions asked by research staff at each PC visit from each PC participant to determine if they had started TB treatment in the preceding 12 months AND criteria used to define self-reported TB. MM/YYYY=Month/Year

Strategy to prepare and analyse the cohort data

| Step 1 | **Generating observation times for each PC visit that took place for each PC participant.**  Because self-reported TB was determined over the 14 months before each PC visit, for each PC participant, an **observation start date**, 14 months before each PC visit was generated. The time between the observation start date for a PC visit, and the date of that PC visit was the observation time for that PC visit, during which the outcome (self-reported TB) was determined.  PC0 observation start date  PC0 visit  PC12 visit date  PC24 visit date  PC36 visit date  PC12 observation start date  PC24 observation start date  PC36 observation start date  Observation time for PC0  Observation time for PC12  Observation time for PC24  Observation time for PC36 |
| --- | --- |
| Step 2 | **Restricting the analysis to the first self-reported TB episode to determine self-reported TB incidence from a “new” TB transmission event**.  Individuals reporting multiple episodes of self-reported TB were explored to understand if these were likely to be treatment starts for TB disease due to new transmission events.  The results of this exploration are shown below.  The total enrolled at PC0 was 38474. TB treatment was self-reported by 628 at any PC visit of whom 55/628 (9%) self-reported TB treatment at >1 PC visit.  Characteristics of 55 PC participants with >1 self-reported TB treatment episode   \| **Characteristic** \| \| **>1 self-reported TB episode** \| \| \| --- \| --- \| --- \| --- \| \| **n/N** \| **%** \| \| Study arm \| A \| 7/55 \| 13% \| \|  \| B \| 26/55 \| 47% \| \|  \| C \| 22/55 \| 40% \| \| Country \| Zambia \| 15/55 \| 27% \| \|  \| South Africa \| 40/55 \| 73% \|   Among these 55 PC participants with >1 self-reported TB treatment episode, 45 (82%) had month and year of TB treatment starts.  If the interval between the two self-reported TB treatment start months/years was ≤14 months, these were considered as starting TB treatment for the same TB episode. This is because TB treatment takes 6-8 months. New TB events following MTB infection take months/years to develop. Therefore 2 self-reported TB treatment starts within 12 months were unlikely to represent TB disease due to new transmission events. They were more likely to represent treatment after lost to follow-up, re-treatment after failure etc. As month of TB treatment start may have been recalled incorrectly a 14-month period between two self-reported TB treatment starts was allowed.   \| **Characteristic** \| \| **n/N** \| **%** \| \| --- \| --- \| --- \| --- \| \| Number of months between TB treatment starts among 34/45 (76%) where interval between self-reported TB treatment start months/years was ≤14 months. 33/34 (97%) reported starting TB treatment at consecutive PC visits. \| same month/year \| 11/34 \| 32% \| \| >0 to ≤2 months \| 9/34 \| 26% \| \| >2 to ≤6 months \| 2/34 \| 6% \| \| >6 to ≤9 months \| 5/34 \| 15% \| \| >9 to ≤12 months \| 6/34 \| 18% \| \| >12 to ≤14 months \| 1/34 \| 3% \| \| Number of months between TB treatment starts among 11/45 (24%) where interval between self-reported TB treatment start months/years was >14 months. 7/11 (64%) did **not** report starting TB treatment at consecutive PC visits \| >14 to ≤18 months \| 2/11 \| 18% \| \| >18 to ≤24 months \| 4/11 \| 36% \| \| >24 months \| 5/11 \| 45% \| \| Arm of n=11 where interval between self-reported TB treatment start months/years was >14 months \| A \| 1/11 \| 9% \| \| B \| 4/11 \| 36% \| \| C \| 6/11 \| 55% \| \| Country of n=11 where duration between self-reported TB treatment start months/years was >14 months \| Zambia \| 4/11 \| 36% \| \| South Africa \| 7/11 \| 64% \|   Among 10/55 (18%) month and year for at least 1 self-reported TB episode was missing. Therefore, interval between treatment starts could not be ascertained. All self-reported TB treatment episodes occurred at consecutive PC visits. For these 10 individuals, the median time between the PC visits at which they self-reported TB treatment was 11.8 months (range 9.5-13.6 months).  The frequency measure of interest is incidence. There were very few repeat self-reported TB events that were likely to represent treatment starts for unique TB episodes. The most plausible estimate of repeat treatment starts for unique TB episodes was 1.8% (11/628 who all had a duration between self-reported TB months/years of >14 months). The maximum value is likely to be 3.3% (21/628, which included the 10 PC participants for whom interval between self-reported TB could not be calculated, but who all reported TB treatment start at consecutive PC visits). As a higher proportion of repeat TB treatment starts were documented in arm C than A, using only the first self-reported TB episode will give a conservative estimate of the impact of the interventions in arms A vs C. |
| Step 3 | **Defining the date of self-reported TB.**  For n=628 individuals who self-reported TB. 587/628 (93%) provided a month and year of treatment start. The day of the month was imputed as 15 for these individuals. 41/628 (7%) did not provide a month and year of TB treatment start. For these individuals, the date of TB treatment start was imputed as the mid-point between two consecutive PC visits, or 7 months before the PC visit where treatment was reported if PC visits were not consecutive. |
| Step 4 | **Defining the date of entry and exit from the cohort.**  The date of entry was the PC0 observation start date that was generated 14 months before the PC0 visit. The date of exit was the last PC visit date if NO self-reported TB. If TB treatment was reported, the date of exit was the date of self-reported TB. |
| Step 5 | **Generating gaps in observation time by calendar year.**  The time between the date of entry and the date of exit from the cohort, was the total follow-up time. Where there were gaps in observation time (e.g. due to missed PC visits), the gap in observation time was determined as the difference between the PC visit date (after which there was a gap) and the observation start date for the subsequent PC visit that took place. All gaps in observation time for each PC participant was generated by the calendar year/s in which the gaps occurred.  0.10  PC0 visit date  PC36 visit date: **date of exit from the cohort**  PC0 observation start date: **date of entry to the cohort**  PC36 observation start date  **No** PC12 visit  **No** PC24 visit  Gap in observation time (between PC0 visit date and PC36 observation start date) calculated and removed from the total follow-up time  Total follow-up time = time between date of entry and date of exit from the cohort |
| Step 6 | **Splitting follow-up time into calendar years and removing the calculated gaps in observation time.**  The total follow-up time generated for each PC participant (time between the date of entry and exit from the cohort) was split into calendar years from 2014 (the first full study year). The calendar periods analysed were 2014, 2015, 2016 and 2017/18 (as follow up in 2018 was only 6 months [PC ended in July 2018], 2017/18 was analysed as one calendar period). Gaps in observation time during which outcome status was unknown, were removed. Gaps were removed according to the calendar year/s in which they occurred.  **2014**  **2015**  **2016**  **2017/18**   - **2**   Gaps in follow up **by calendar year** removed. |
| Step 7 | **Assigning HIV status for each calendar year and running sensitivity analysis on HIV status.**  **A:** The HIV status at each PC visit was determined using blood HIV testing done at the PC visit. This HIV blood test result was used to define the primary HIV endpoint of the trial.  **2017/18**  **2014**  **2015**  **2016**  PC0-visit in 2014:  HIV negative  PC12-visit in 2015:  HIV status unknown  PC24-visit in 2016:  HIV status unknown  PC36 visit in 2017:  HIV positive  **B:** The HIV status at the PC visit was assumed to be the HIV status for the whole calendar year in which the PC visit took place and therefore, for the observation time contributed by the PC participant for that calendar year.  If HIV status in a calendar year to which the PC participant contributed observation time was HIV positive, all subsequent calendar years to which the PC participant contributed observation time where HIV status was unknown, was imputed as HIV positive.  If HIV status in a calendar year in which the PC participant contributed observation time was HIV negative, all preceding calendar years to which the PC participant contributed observation time where HIV status was unknown, was imputed as HIV negative.  Analyses stratified by calendar year and HIV status were conducted based on this HIV status assignment.  **2017/18**  **2014**  **2015**  **2016**  HIV status for observation time in 2014 is HIV negative  HIV status for observation time in 2015 is unknown  HIV status for observation time in 2016 is unknown  HIV status for observation time in 2017/18 is HIV positive  **C:** Where HIV status in the year preceding a HIV positive result was unknown (i.e. in 2016 in this example), a first sensitivity analysis was conducted assuming the missing HIV status was positive.  **2017/18**  **2014**  **2015**  **2016**  HIV status for observation time in 2014 is HIV negative  HIV status for observation time in 2015 is unknown  **On sensitivity analysis HIV status first assumed to be HIV positive in 2016**  HIV status for observation time in 2017/18 is HIV positive  **D:** Where HIV status in the year preceding a HIV positive result was unknown (i.e. in 2016 in this example), a second sensitivity analysis was conducted assuming the missing HIV status was negative.  **2017/18**  **2014**  **2015**  **2016**  HIV status for observation time in 2014 is HIV negative  HIV status for observation time in 2015 is unknown  **On sensitivity analysis HIV status then assumed to be HIV negative in 2016**  HIV status for observation time in 2017/18 is HIV positive |
| Step 8 | **Summary of the characteristics of PC participants contributing person time to the cohort analysis, by calendar year and study arm**  For 2014, 2015, 2016 and 2017/18 separately. By study arm (A, B, C, and total) for each calendar year  Variables summarised   - Country – Zambia, South Africa (defined at PC0) - Sex – Male, Female (defined at PC0) - Age/years – in years (defined at PC0) stratified into ~5year age groups (18-24, 25-29, 30-34, 35-39, 40-44) - HIV-status – as defined for each calendar year (step 7B)   Data showed losses to follow up over calendar years by study arm and characteristics of those who were included in the analysis over calendar years by study arm.  Data were stratified by HIV status**,** for PLHIV and those HIV negative separately, by calendar year and study arm.  For 2014, 2015, 2016 and 2017/18 separately  By study arm (A, B, C, and total) for each calendar year  Variables summarised   - Country – Zambia, South Africa (defined at PC0) - Sex – Male, Female (defined at PC0) - Age/years – in years (defined at PC0) stratified into ~5year age groups (18-24, 25-29, 30-34, 35-39, 40-44) |
| Step 9 | **The number of events, total person years and incidence rate (per 100 person years) of self-reported TB**  For each community, data were summarised for each calendar year (2014, 2015, 2016 and 2017/18) separately.  Within a calendar year, communities were stratified by triplet and arm.  Where there are no self-reported TB events for a community – 0.5 was added to the numerator to compute a rate. This was needed to compute geometric means, for which rates were multiplied and the nth root of the multiplied value taken.  The overall incidence rate by arm (A, B and C) was computed as the geometric mean of the estimated incidence rates for the 7 communities in each study arm. Geometric means were used to reduce skewness.  Data were also be summarised by HIV status (for people who are HIV positive and HIV negative separately) for each calendar year. HIV status used the assignment generated in Step 7B. Sensitivity analysis explored assigning HIV status as described in Step 7 C-D. |
| Step 10 | **Cross-arm comparison of incidence rate of self-reported TB**  Arm A versus B and Arm B versus C separately.  For each calendar year (2014, 2015, 2016 and 2017/18) separately.  Overall and for PLHIV.  Stage 1: Poisson regression was used to adjust for confounding variables at the individual level for each country separately.  Covariates added:   - Triplet – primary model for analysis among PLHIV - Triplet + HIV status – primary model for overall analysis - Triplet + age#sex – over-parameterised model for overall analysis and analysis among PLHIV (insufficient events in later calendar years for this model). This additional analysis was carried out to check the effect of adjusting for age and sex on the point estimate of the rate ratio. - Triplet + age#sex + HIV status - over-parameterised model for overall analysis (insufficient events in later calendar years for this model). This additional analysis was carried out to check the effect of adjusting for age, sex, and HIV status on the point estimate of the rate ratio.   Study arm was NOT included in stage 1.  For each PC participant a fitted value of the outcome (incident self-reported TB) was predicted from the model. These fitted values were summed for each community, to get E, the expected number with incident self-reported TB for each community, after adjusting for covariates, assuming null intervention effects. The ratio residual for each community was calculated as the Observed number of incident self-reported TB events (O, with 0.5 added if no events were observed), divided by the Expected number of incident self-reported TB events (E).  Stage 2: A two-way analysis of variance was carried out on the log(O/E) (log ratio-residuals), with matched triplet and study arm as factors. The test statistic was the estimated difference in means of log(O/E) between study arms, with two-sided p-values and 95% confidence intervals computed using the t-distribution. The corresponding rate ratios and 95% confidence interval for the comparison of Arms A and C, and Arms B and C, was calculated with exponentiation. A log transformation is generally used for the analysis of ratio measures of effect (e.g. rate ratios, risk ratios, prevalence ratios) because they are often positively skewed.  NB: models with age and sex added gave similar point estimates of the rate ratios, as models without age and sex. |

Strategy to prepare and analyse the cross-sectional data

| Step 1 | **Generating the samples for analysis.**  Each PC visit – PC0, PC12, PC24, and PC36 – was treated as an independent cross-sectional sample.  The denominator included all PC participants seen at that PC visit |
| --- | --- |
| Step 2 | **Self-reported TB.**  Included all PC participants meeting the case definition of self-reported TB. All episodes of self-reported TB (n=686 which included repeat episodes) were analysed. |
| Step 3 | **Assigning HIV status for each PC visits.**  The HIV status at each PC visit was determined using blood HIV testing done at the PC visit. This HIV blood test result was used to define the primary HIV endpoint of the trial. |
| Step 4 | **Summary of the number of PC participants meeting the self-reported TB case definition.**  For each PC visit - PC0, PC12, PC24, and PC36 – separately  Overall and stratified by country and HIV status, for each PC visit |
| Step 5 | **Summary of the characteristics of PC participants seen at PC0, PC12, PC24 and PC36, by study arm.**  For each PC visit - PC0, PC12, PC24, and PC36 – separately  Variables to be summarised   - Country – Zambia, South Africa (defined at PC0) - Sex – Male, Female (defined at PC0) - Age/years – in years stratified into ~5year age groups (18-24, 25-29, 30-34, 35-39, 40-max). Age determined at PC0. Age at each subsequent PC visits, was based on adding 1 to age. - HIV-status – as defined for each PC visit.   Data showed losses to follow up at each PC visit by study arm and characteristics of those included in the analysis at each PC visit by study arm.  Results were stratified by HIV status, for PLHIV and those HIV negative separately, for each PC visit and by study arm.  Variables to be summarised:   - Country – Zambia, South Africa (defined at PC0) - Sex – Male, Female (defined at PC0) - Age/years – in years stratified into ~5year age groups (18-24, 25-29, 30-34, 35-39, 40-max). Age determined at PC0. Age at each subsequent PC visits, was based on adding 1 to age. |
| Step 6 | **The number of events, total number of PC participants and proportion with self-reported TB**  For each community, data were summarised for each PC visit - PC0, PC12, PC24, and PC36 – separately.  For each PC visit, communities were stratified by triplet and arm.  Where there are no self-reported TB events for a community – 0.5 was added to the numerator to compute a proportion. This was needed to generate geometric means, for which proportions were multiplied and the nth root of the multiplied value taken.  The overall proportion by arm (A, B and C) was computed as the geometric mean of the estimated proportions for the 7 communities in each study arm. Geometric means were used to reduce skewness.  Data were summarised by HIV status (for people who are HIV positive and HIV negative separately) for each PC visit. |
| Step 7 | **Cross-arm comparison of proportion self-reporting TB**  Arm A versus B and Arm B versus C separately  For each PC visit – PC0, PC12, PC24, and PC36 - separately.  Overall and for PLHIV  Stage 1: Logistic regression was used to adjust for confounding variables at the individual level for each country separately.  Covariates to be added:   - Triplet – primary model for analysis among PLHIV - Triplet + HIV status – primary model for overall analysis - Triplet + age#sex– over-parameterised model for overall analysis and analysis among PLHIV (insufficient events in later calendar years for this model). This additional analysis was carried out to check the effect of adjusting for age and sex on the point estimate of the prevalence ratio. - Triplet + age#sex + HIV status - over-parameterised model for overall analysis (insufficient events in later calendar years for this model). This additional analysis was carried out to check the effect of adjusting for age, sex, and HIV status on the point estimate of the prevalence ratio.   Study arm was NOT included in stage 1.  For each PC participant a fitted value of the outcome (self-reported TB) was predicted from the model. These fitted values were summed for each community, to get E, the expected number with self-reported TB for each community, after adjusting for covariates, assuming null intervention effects. The ratio residual for each community was calculated as the Observed number of self-reported TB events (O, with 0.5 added if no events were observed), divided by the Expected number of self-reported TB events (E).  Stage 2: A two-way analysis of variance was carried out on the log(O/E) (log ratio-residuals), with matched triplet and study arm as factors. The test statistic was the estimated difference in means of log(O/E) between study arms, with two-sided p-values and 95% confidence intervals computed using the t-distribution. The corresponding prevalence ratios and 95% confidence interval for the comparison of Arms A and C, and Arms B and C, was calculated with exponentiation. A log transformation is generally used for the analysis of ratio measures of effect (e.g. rate ratios, risk ratios, prevalence ratios) because they are often positively skewed.  NB: models with age and sex added gave similar point estimates of the prevalence ratios, as models without age and sex. |

[1] Ayles H, Muyoyeta M, Du Toit E, et al. Effect of household and community interventions on the burden of tuberculosis in southern Africa: the ZAMSTAR community-randomised trial. Lancet 2013; 382(9899): 1183-94

[2] Eldridge S, Kerry S. A practical guide to cluster randomised trials in health services research. Chichester, United Kingdom: John Wiley, 2012.

[3] Hayes RJ, Moulton LH. Cluster randomised trials. 2nd ed. Boca Raton, FL:CRC Press, 2017
