# Supplementary material for: Incidence of self-reported tuberculosis treatment with community-wide universal testing and treatment for HIV and tuberculosis screening in Zambia and South Africa: A planned analysis of the HPTN 071 (PopART) cluster-randomised trial
Source: PLoS Med. 2024 May 31;21(5):e1004393. doi: 10.1371/journal.pmed.1004393 (PMC11142425; doi:10.1371/journal.pmed.1004393)
Supplement: S5 Appendix — (DOCX) [file pmed.1004393.s005.docx]

**S5 Appendix**

|  |  | **PC0** | | | | **PC12** | | | | **PC24** | | | | **PC36** | | | |
| --- | --- | --- | --- | --- | --- | --- | --- | --- | --- | --- | --- | --- | --- | --- | --- | --- | --- |
|  |  | **A** | **B** | **C** | **Total** | **A** | **B** | **C** | **Total** | **A** | **B** | **C** | **Total** | **A** | **B** | **C** | **Total** |
|  |  |  |  |  |  |  |  |  |  |  |  |  |  |  |  |  |  |
| Total seen |  | 12,671* | 13,404* | 12,399* | 38,474* | 8234* (65%)^¶^ | 8572*  (64%)^¶^ | 8484* (68%)^¶^ | 25290* (66%)^¶^ | 6938* (55%)^¶^ | 7873* (59%)^¶^ | 6867* (55%)^¶^ | 21678* (56%)^¶^ | 6623* (52%)^¶^ | 7416* (55%)^¶^ | 6383* (51%)^¶^ | 20422* (53%)^¶^ |
|  |  |  |  |  |  |  |  |  |  |  |  |  |  |  |  |  |  |
| Country | Zambia | 6,500 (51%) | 6,433 (48%) | 6,791 (55%) | 19,724 (51%) | 4067 (49%) | 3893 (45%) | 4371 (52%) | 12331 (49%) | 3530 (51%) | 3760 (48%) | 3637 (53%) | 10927 (50%) | 3539 (53%) | 3720 (50%) | 3686 (58%) | 10945 (54%) |
|  | SA | 6,171 (49%) | 6,971 (52%) | 5,608 (45%) | 18,750 (49%) | 4167 (51%) | 4679 (55%) | 4113 (48%) | 12959 (51%) | 3408 (49%) | 4113 (52%) | 3230 (47%) | 10751 (50%) | 3084 (47%) | 3696 (50%) | 2697 (42%) | 9477 (46%) |
|  |  |  |  |  |  |  |  |  |  |  |  |  |  |  |  |  |  |
| Sex | Male | 3595 (28%) | 3906 (29%) | 3701 (30%) | 11202 (29%) | 2150 (26%) | 2289 (27%) | 2381 (28%) | 6820 (27%) | 1753 (25%) | 2122 (27%) | 1930 (28%) | 5805 (27%) | 1654 (25%) | 1967 (27%) | 1775 (28%) | 5396 (26%) |
|  | Female | 9042 (71%) | 9458 (71%) | 8639 (70%) | 27139 (71%) | 6084 (74%) | 6281 (73%) | 6103 (72%) | 18468 (73%) | 5185 (75%) | 5751 (73%) | 4937 (72%) | 15873 (73%) | 4969 (75%) | 5449 (73%) | 4607 (72%) | 15025 (74%) |
|  | Missing | 34  (<1%) | 40  (<1%) | 59  (<1%) | 133 (<1%) | 0  (0%) | 2  (<1%) | 0  (0%) | 2  (<1%) | 0  (0%) | 0  (0%) | 0  (0%) | 0  (0%) | 0  (0%) | 0  (0%) | 1  (<1%) | 1  (<1%) |
|  |  |  |  |  |  |  |  |  |  |  |  |  |  |  |  |  |  |
| Age (years)^†^ | 18-24 | 5065 (40%) | 5179 (39%) | 4981 (40%) | 15225 (40%) | 2791 (34%) | 2801 (33%) | 2811 (33%) | 8403 (33%) | 1959 (28%) | 2176 (28%) | 1889 (28%) | 6024 (28%) | 1517 (23%) | 1671 (22%) | 1400 (22%) | 4588 (22%) |
|  | 25-29 | 2781 (22%) | 2881 (21%) | 2608 (21%) | 8270 (22%) | 1825 (22%) | 1777 (21%) | 1830 (21%) | 5432 (21%) | 1517 (22%) | 1661 (21%) | 1450 (21%) | 4628 (21%) | 1499 (23%) | 1640 (22%) | 1406 (22%) | 4545 (22%) |
|  | 30-34 | 2147 (17%) | 2289 (17%) | 2080 (17%) | 6516 (17%) | 1456 (18%) | 1600 (19%) | 1499 (18%) | 4555 (18%) | 1343 (19%) | 1538 (19%) | 1314 (19%) | 4195 (19%) | 1346 (20%) | 1449 (20%) | 1250 (19%) | 4045 (20%) |
|  | 35-39 | 1464 (12%) | 1713 (13%) | 1558 (13%) | 4735 (12%) | 1119 (13%) | 1236 (14%) | 1253 (15%) | 3608 (14%) | 1015 (15%) | 1166 (15%) | 1083 (16%) | 3264 (15%) | 1034 (16%) | 1150 (16%) | 1064 (17%) | 3248 (16%) |
|  | 40/max^†^ | 1179 (9%) | 1302 (10%) | 1109 (9%) | 3590 (9%) | 1043 (13%) | 1156 (13%) | 1091 (13%) | 3290 (13%) | 1104 (16%) | 1332 (17%) | 1129 (16%) | 3565 (16%) | 1226 (18%) | 1506 (20%) | 1261 (20%) | 3993 (20%) |
|  | Missing | 35  (<1%) | 40  (<1%) | 63  (<1%) | 138 (<1%) | 0  (0%) | 2  (<1%) | 0  (0%) | 2  (<1%) | 0  (0%) | 0  (0%) | 2  (<1%) | 2  (<1%) | 1  (<1%) | 0  (0%) | 2  (<1%) | 3  (<1%) |
|  |  |  |  |  |  |  |  |  |  |  |  |  |  |  |  |  |  |
| HIV status^‡^ | Positive | 2583 (20%) | 2734 (20%) | 2687 (22%) | 8004 (21%) | 1661 (20%) | 1660 (19%) | 1765 (21%) | 5086 (20%) | 1459 (21%) | 1608 (21%) | 1512 (22%) | 4579 (21%) | 1549 (23%) | 1637 (22%) | 1572 (25%) | 4758 (23%) |
|  | Negative | 9594 (76%) | 10235 (77%) | 9301 (75%) | 29130 (76%) | 5781 (70%) | 6210 (73%) | 5678 (67%) | 17669 (70%) | 4931 (71%) | 5691 (72%) | 4672 (68%) | 15294 (71%) | 4873 (74%) | 5587 (75%) | 4651 (73%) | 15111 (74%) |
|  | ND | 494  (4%) | 435  (3%) | 411  (3%) | 1340 (3%) | 792 (10%) | 702  (8%) | 1041 (12%) | 2535 (10%) | 548  (8%) | 574  (7%) | 683 (10%) | 1805 (8%) | 201  (3%) | 192  (3%) | 160  (2%) | 553  (3%) |

**Table: Characteristics of Population Cohort participants enrolled at PC0 and follow-up at PC12, PC24 and PC36 respectively from all 21 HPTN 071 (PopART) communities: overall and by study arm**

PC=population cohort; SA=South Africa; ND=not determined. *Denominator for all column percentages shown in the column (unless otherwise indicated); ^¶^Denominator for this proportion was the number seen at PC0 in each study arm and in total; ^†^Age determined at PC0. Age at each subsequent PC-visit, based on adding 1 to the age the PC-participant would have been at the preceding PC-visit, starting at PC0. Upper limits of age are PC0=44 years PC12=45 years, PC24=46 years, and PC36=47years; ^‡^based on laboratory HIV-testing
