# Supplementary material for: Incidence of self-reported tuberculosis treatment with community-wide universal testing and treatment for HIV and tuberculosis screening in Zambia and South Africa: A planned analysis of the HPTN 071 (PopART) cluster-randomised trial
Source: PLoS Med. 2024 May 31;21(5):e1004393. doi: 10.1371/journal.pmed.1004393 (PMC11142425; doi:10.1371/journal.pmed.1004393)
Supplement: S6 Appendix — (DOCX) [file pmed.1004393.s006.docx]

**S6 Appendix**

Total enrolled at PC0 = 38,474 from all 21 communities

**Arm A (7 communities; 12,671 [33%])**

**Arm B (7 communities; 13,404 [35%])**

**Arm C (7 communities; 12,399 [32%])**

**PC12**

8,234 (65%) Completed visit

2,406 (19%) Missed visit

2,031 (16%) Terminated^¶^

- 416 (21%) Refused
- 1,357 (67%) Relocated
- 65 (3%) Incapacitated/hospitalised
- 88 (4%) Died
- 105 (5%) Other

**PC12**

8,572 (64%) Completed visit

3,193 (24%) Missed visit

1,639 (12%) Terminated^¶^

- 304 (18%) Refused
- 1,026 (63%) Relocated
- 141 (9%) Incapacitated/hospitalised
- 81 (5%) Died
- 87 (5%) Other

**PC12**

8,484 (68%) Completed visit

2,394 (19%) Missed visit

1,521 (12%) Terminated^¶^

- 228 (15%) Refused
- 1,117 (74%) Relocated
- 49 (3%) Incapacitated/hospitalised
- 68 (4%) Died
- 58 (4%) Other

**PC24 (10,640 [84% of total*] in f/u)**

6,938 (65%; 55% of total*) Completed visit

2,239 (21%) Missed visit

1,463 (14%) Terminated^¶^

- 379 (26%) Refused
- 938 (64%) Relocated
- 3 (<1%) Incapacitated/hospitalised
- 45 (3%) Died
- 93 (6%) Other

**PC24 (11,765 [88% of total*] in f/u)**

7,873 (67%; 59% of total*) Completed visit

2,473 (21%) Missed visit

1,419 (12%) Terminated^¶^

- 338 (24%) Refused
- 915 (66%) Relocated
- 1 (<1%) Incapacitated/hospitalised
- 70 (5%) Died
- 66 (5%) Other

**PC24 (10,878 [88% of total*] in f/u)**

6,867 (63%; 55% of total*) Completed visit

2,311 (21%) Missed visit

1,700 (16%) Terminated^¶^

- 386 (23%) Refused
- 1,117 (66%) Relocated
- 2 (<1%) Incapacitated/hospitalised
- 66 (4%) Died
- 121 (7%) Other

**PC36 (9,177 [72% of total*] in f/u)**

6,623 (72%; 52% of total*) Completed visit

2,554 (28%) Terminated^¶^

- 473 (19%) Refused
- 1,258 (49%) Relocated
- 4 (<1%) Incapacitated/hospitalised
- 57 (2%) Died
- 155 (6%) Other
- 607 (24%) Not located

**PC36 (10,346 [77% of total*] in f/u)**

7,416 (72%; 55% of total*) Completed visit

2,930 (28%) Terminated^¶^

- 480 (16%) Refused
- 1,520 (52%) Relocated
- 6 (<1%) Incapacitated/hospitalised
- 49 (2%) Died
- 136 (5%) Other
- 739 (25%) Not located

**PC36 (9,178 [74% of total*] in f/u)**

6,383 (70%; 51% of total*) Completed visit

2,795 (30%) Terminated^¶^

- 686 (25%) Refused
- 1,462 (52%) Relocated
- 7 (<1%) Incapacitated/hospitalised
- 51 (2%) Died
- 136 (5%) Other
- 453 (16%) Not located

**Figure: Consort flow diagram showing the Population Cohort participants from all 21 HPTN 071 (PopART) communities that contributed to the cross-sectional analysis**

PC=Population Cohort; f/u=follow-up; ^¶^1 person with unknown termination reason at PC12; 42 people with unknown termination reason at PC24 (5 [<1% in arm A; 29 [2%] in arm B; and 8 [<1%] in arm C); *denominator was the total enrolled in that arm at PC0
