# Supplementary material for: Incidence of self-reported tuberculosis treatment with community-wide universal testing and treatment for HIV and tuberculosis screening in Zambia and South Africa: A planned analysis of the HPTN 071 (PopART) cluster-randomised trial
Source: PLoS Med. 2024 May 31;21(5):e1004393. doi: 10.1371/journal.pmed.1004393 (PMC11142425; doi:10.1371/journal.pmed.1004393)
Supplement: S7 Appendix — (DOCX) [file pmed.1004393.s007.docx]

**S7 Appendix**

|  |  | **All follow-up visits (PC12-36)** | | **PC12 visit** | | **PC24 visit** | | **PC36 visit** | |
| --- | --- | --- | --- | --- | --- | --- | --- | --- | --- |
|  |  | **Not seen** | **Seen at least once** | **Not seen** | **Seen** | **Not seen** | **Seen** | **Not seen** | **Seen** |
|  |  |  |  |  |  |  |  |  |  |
| Total |  | 10,526 (27%)^¶^ | 27,948 (73%)^¶^ | 13,184 (34%)^¶^ | 25,290 (66%)^¶^ | 16,796 (44%)^¶^ | 21,678 (56%)^¶^ | 18,052 (47%)^¶^ | 20,422 (53%)^¶^ |
|  |  |  |  |  |  |  |  |  |  |
| Country | Zambia | 5,670 (54%) | 14,054 (50%) | 7,393 (56%) | 12,331 (49%) | 8,797 (52%) | 10,927 (50%) | 4,096 (49%) | 10,945 (54%) |
|  | SA | 4,856 (46%) | 13,894 (50%) | 5,791 (44%) | 12,959 (51%) | 7,999 (48%) | 10,751 (50%) | 9,273 (51%) | 9,477 (46%) |
|  |  |  |  |  |  |  |  |  |  |
| Sex | Male | 3,460 (33%) | 7,742 (28%) | 4,382 (34%) | 6,820 (27%) | 5,397 (32%) | 5,805 (27%) | 5,806 (32%) | 5,396 (26%) |
|  | Female | 6,936 (67%) | 20,203 (72%) | 8,671 (66%) | 18,468 (73%) | 11,266 (68%) | 15,873 (73%) | 12,114 (68%) | 15,025 (74%) |
|  |  |  |  |  |  |  |  |  |  |
| Age (years)^†^ | 18-24 | 4,582 (44%) | 10,643 (38%) | 5,693 (44%) | 9,532 (38%) | 7,244 (43%) | 7,981 (37%) | 7,748 (43%) | 7,477 (37%) |
|  | 25-29 | 2,465 (24%) | 5,805 (21%) | 3,012 (23%) | 5,258 (21%) | 3,894 (23%) | 4,376 (20%) | 4,133 (23%) | 4,137 (20%) |
|  | 30-34 | 1,618 (16%) | 4,898 (18%) | 2,091 (16%) | 4,425 (18%) | 2,590 (16%) | 3,926 (18%) | 2,857 (16%) | 3,659 (18%) |
|  | 35-39 | 1,000 (10%) | 3,735 (13%) | 1,304 (10%) | 3,431 (14%) | 1,709 (10%) | 3,026 (14%) | 1,847 (10%) | 2,888 (14%) |
|  | 40/max | 729 (7%) | 2,861 (10%) | 948 (7%) | 3,431 (10%) | 1,223 (7%) | 2,367 (11%) | 1,332 (7%) | 2,258 (11%) |
|  |  |  |  |  |  |  |  |  |  |
| HIV status^‡^ | Negative | 7,630 (76%) | 21,500 (79%) | 9,642 (77%) | 19,488 (79%) | 12,390 (77%) | 16,740 (79%) | 13,355 (77%) | 15,775 (79%) |
|  | Positive | 2,377 (24%) | 5,627 (21%) | 2,906 (23%) | 5,098 (21%) | 3,667 (23%) | 4,337 (21%) | 3,908 (23%) | 4,096 (21%) |

**Table: Characteristics of individuals not seen and seen (at least once during follow up and at each follow up PC visit [PC12, PC24 AND PC36, respectively]).**

PC=population cohort; SA=South Africa; ^†^age at PC0; ^‡^HIV-status at PC0; ^¶^column percentages shown
