# Supplementary material for: Incidence of self-reported tuberculosis treatment with community-wide universal testing and treatment for HIV and tuberculosis screening in Zambia and South Africa: A planned analysis of the HPTN 071 (PopART) cluster-randomised trial
Source: PLoS Med. 2024 May 31;21(5):e1004393. doi: 10.1371/journal.pmed.1004393 (PMC11142425; doi:10.1371/journal.pmed.1004393)
Supplement: S8 Appendix — (DOCX) [file pmed.1004393.s008.docx]

**S8 Appendix**

|  |  | **Characteristics, by PC round, of people who were HIV-positive at each PC round** | | | | | | | | | | | | | | | |
| --- | --- | --- | --- | --- | --- | --- | --- | --- | --- | --- | --- | --- | --- | --- | --- | --- | --- |
|  |  | **PC0** | | | | **PC12** | | | | **PC24** | | | | **PC36** | | | |
|  |  | **A** | **B** | **C** | **Total** | **A** | **B** | **C** | **Total** | **A** | **B** | **C** | **Total** | **A** | **B** | **C** | **Total** |
| Total seen |  | 2,583*  (32%)^¶^ | 2,734*  (34%)^¶^ | 2,687*  (34%)^¶^ | 8,004*  (100%)^¶^ | 1,661*  (33%)^¶^ | 1,660*  (33%)^¶^ | 1,765*  (34%)^¶^ | 5,086*  (100%)^¶^ | 1,459*  (32%)^¶^ | 1,608*  (35%)^¶^ | 1,512*  (33%)^¶^ | 4,579*  (100%)^¶^ | 1,549*  (33%)^¶^ | 1,637*  (34%)^¶^ | 1,572*  (33%)^¶^ | 4,758*  (100%)^¶^ |
|  |  |  |  |  |  |  |  |  |  |  |  |  |  |  |  |  |  |
| Country | Zambia | 1254 (49%) | 1396 (51%) | 1395 (52%) | 4045 (51%) | 860 (52%) | 882 (53%) | 949 (54%) | 2691 (53%) | 788 (54%) | 917 (57%) | 838 (55%) | 2543 (56%) | 862 (56%) | 950 (58%) | 920 (59%) | 2732 (57%) |
|  | SA | 1329 (51%) | 1338 (49%) | 1292 (48%) | 3959 (49%) | 801 (48%) | 778 (47%) | 816 (46%) | 2395 (47%) | 671 (46%) | 691 (43%) | 674 (45%) | 2036 (44%) | 687 (44%) | 687 (42%) | 652 (41%) | 2026 (43%) |
|  |  |  |  |  |  |  |  |  |  |  |  |  |  |  |  |  |  |
| Sex | Male | 414 (16%) | 406 (15%) | 430 (16%) | 1250 (16%) | 239 (14%) | 202 (12%) | 265 (15%) | 706 (14%) | 205 (14%) | 207 (13%) | 221 (15%) | 633 (14%) | 216 (14%) | 217 (13%) | 235 (15%) | 668 (14%) |
|  | Female | 2160 (84%) | 2324 (85%) | 2241 (83%) | 6725 (84%) | 1422 (86%) | 1458 (88%) | 1500 (85%) | 4380 (86%) | 1254 (86%) | 1401 (87%) | 1291 (85%) | 3946 (86%) | 1333 (86%) | 1420 (87%) | 1337 (85%) | 4090 (86%) |
|  | Missing | 9  (<1%) | 4  (<1%) | 16  (<1%) | 29  (<1%) | 0  (0%) | 0  (0%) | 0  (0%) | 0  (0%) | 0  (0%) | 0  (0%) | 0  (0%) | 0  (0%) | 0  (0%) | 0  (0%) | 0  (0%) | 0  (0%) |
|  |  |  |  |  |  |  |  |  |  |  |  |  |  |  |  |  |  |
| Age (years) ^†^ | 18-24 | 457 (18%) | 522 (19%) | 501 (19%) | 1480 (18%) | 225 (13%) | 232 (14%) | 274 (16%) | 731 (14%) | 167 (11%) | 180 (11%) | 186 (12%) | 533 (12%) | 157 (10%) | 164 (10%) | 157 (10%) | 478 (10%) |
|  | 25-29 | 606 (23%) | 600 (22%) | 600 (22%) | 1806 (23%) | 351 (21%) | 315 (19%) | 341 (19%) | 1007 (20%) | 252 (17%) | 296 (18%) | 247 (16%) | 795 (17%) | 262 (17%) | 293 (18%) | 260 (17%) | 815 (17%) |
|  | 30-34 | 623 (24%) | 674 (25%) | 625 (23%) | 1922 (24%) | 398 (24%) | 412 (25%) | 381 (21%) | 1191 (23%) | 361 (25%) | 406 (25%) | 326 (21%) | 1093 (24%) | 366 (24%) | 352 (21%) | 326 (21%) | 1044 (22%) |
|  | 35-39 | 506 (20%) | 568 (21%) | 555 (21%) | 1629 (20%) | 361 (22%) | 377 (23%) | 419 (24%) | 1157 (23%) | 311 (21%) | 348 (22%) | 389 (26%) | 1048 (23%) | 331 (21%) | 358 (22%) | 377 (24%) | 1066 (22%) |
|  | 40/max^†^ | 382 (15%) | 366 (13%) | 389 (14%) | 1137 (14%) | 326 (20%) | 324 (20%) | 350 (20%) | 1000 (20%) | 368 (25%) | 378 (23%) | 364 (24%) | 1110 (24%) | 432 (28%) | 470 (29%) | 452 (29%) | 1354 (28%) |
|  | missing | 9  (<1%) | 4  (<1%) | 17  (<1%) | 30  (<1%) | 0  (0%) | 0  (0%) | 0  (0%) | 0  (0%) | 0  (0%) | 0  (0%) | 0  (0%) | 0  (0%) | 1  (<1%) | 0  (0%) | 0  (0%) | 1  (<1%) |
|  |  |  |  |  |  |  |  |  |  |  |  |  |  |  |  |  |  |
|  |  | **Characteristics, by calendar year, of people who were HIV-positive at each calendar year, who contributed person time to the cohort analysis during that specific calendar year** | | | | | | | | | | | | | | | |
|  |  | **2014** | | | | **2015** | | | | **2016** | | | | **2017/18** | | | |
|  |  | **A** | **B** | **C** | **Total** | **A** | **B** | **C** | **Total** | **A** | **B** | **C** | **Total** | **A** | **B** | **C** | **Total** |
| Total seen |  | 2071*  (32%)^¶^ | 2215 *  (35%)^¶^ | 2099*  (33%)^¶^ | 6,385*  (100%)^¶^ | 1884*  (32%)^¶^ | 1943*  (33%)^¶^ | 2047*  (35%)^¶^ | 5874*  (100%)^¶^ | 1707*  (32%)^¶^ | 1775*  (33%)^¶^ | 1854*  (35%)^¶^ | 5336*  (100%)^¶^ | 1620*  (32%)^¶^ | 1728*  (35%)^¶^ | 1649*  (33%)^¶^ | 4997*  (100%)^¶^ |
|  |  |  |  |  |  |  |  |  |  |  |  |  |  |  |  |  |  |
| Country | Zambia | 980 (47%) | 1147 (52%) | 1094 (52%) | 3221 (50%) | 924 (49%) | 1026 (53%) | 1063 (52%) | 3013 (51%) | 889 (52%) | 962 (54%) | 989 (53%) | 2840 (53%) | 878 (54%) | 975 (56%) | 956 (58%) | 2809 (56%) |
|  | SA | 1091 (53%) | 1068 (48%) | 1005 (48%) | 3164 (50%) | 960 (51%) | 917 (47%) | 984 (48%) | 2861 (49%) | 818 (48%) | 813 (46%) | 865 (47%) | 2496 (47%) | 742 (46%) | 753 (44%) | 693 (42%) | 2188 (44%) |
|  |  |  |  |  |  |  |  |  |  |  |  |  |  |  |  |  |  |
| Sex | Male | 305 (15%) | 321 (14%) | 320 (15%) | 946 (15%) | 278 (15%) | 252 (13%) | 314 (15%) | 844 (14%) | 239 (14%) | 233 (13%) | 275 (15%) | 747 (14%) | 232 (14%) | 221 (13%) | 240 (15%) | 693 (14%) |
|  | Female | 1757 (85%) | 1890 (85%) | 1763 (84%) | 5410 (85%) | 1606 (85%) | 1691 (87%) | 1,733 (85%) | 5030 (86%) | 1468 (86%) | 1542 (87%) | 1579 (85%) | 4589 (86%) | 1388 (86%) | 1507 (87%) | 1,409 (85%) | 4,304 (86%) |
|  | Missing | 9  (<1%) | 4  (<1%) | 16  (<1%) | 29  (<1%) | 0  (0%) | 0  (0%) | 0  (0%) | 0  (0%) | 0  (0%) | 0  (0%) | 0  (0%) | 0  (0%) | 0  (0%) | 0  (0%) | 0  (0%) | 0  (0%) |
|  |  |  |  |  |  |  |  |  |  |  |  |  |  |  |  |  |  |
| Age/years^ᶲ^ | 18-24 | 352  (17%) | 410  (19%) | 381  (18%) | 1143  (18%) | 310 (17%) | 349 (18%) | 390 (19%) | 1049 (18%) | 310 (18%) | 338 (19%) | 357 (19%) | 1005 (19%) | 321 (20%) | 371 (21%) | 339 (21%) | 1031 (21%) |
|  | 25-29 | 487  (24%) | 484 (22%) | 464  (22%) | 1435  (23%) | 416 (22%) | 396 (20%) | 425 (21%) | 1237 (21%) | 367 (21%) | 338 (19%) | 383 (21%) | 1088 (21%) | 343 (21%) | 337 (20%) | 327 (20%) | 1007 (20%) |
|  | 30-34 | 505 (24%) | 548 (25%) | 495 (24%) | 1548 (24%) | 461 (24%) | 506 (26%) | 477 (23%) | 1444 (25%) | 407 (24%) | 451 (26%) | 436 (24%) | 1294 (24%) | 381 (24%) | 412 (24%) | 369 (22%) | 1162 (23%) |
|  | 35-39 | 417 (20%) | 469 (21%) | 430 (20%) | 1316 (21%) | 400 (21%) | 414 (21%) | 448 (22%) | 1262 (21%) | 355 (21%) | 380 (21%) | 397 (21%) | 1132 (21%) | 328 (20%) | 353 (20%) | 362 (22%) | 1043 (21%) |
|  | 40/max | 301 (15%) | 300 (13%) | 312 (15%) | 913 (14%) | 297 (16%) | 278 (15%) | 307 (15%) | 882 (15%) | 268 (16%) | 268 (15%) | 281 (15%) | 817 (15%) | 246 (15%) | 255 (15%) | 252 (15%) | 753 (15%) |
|  | Missing | 9  (<1%) | 4  (<1%) | 17  (<1%) | 30  (<1%) | 0  (0%) | 0  (0%) | 0  (0%) | 0  (0%) | 0  (0%) | 0  (0%) | 0  (0%) | 0  (0%) | 1  (<1%) | 0  (0%) | 0  (0%) | 1  (<1%) |

**Table: Characteristics of Population Cohort participants from all 21 HPTN 071 (PopART) communities, who were HIV-positive (based on laboratory HIV-testing) and contributed to the cross-sectional and cohort analysis: overall and by study arm**

PC=population cohort; SA=South Africa; All percentages rounded to the nearest whole number; *Denominator for all column percentages shown in the column (unless otherwise indicated); ^¶^Denominator for this proportion was the total number seen (row percentage); ^†^Age determined at PC0. Age at each subsequent PC-visit, based on adding 1 to the age the PC-participant would have been at the preceding PC-visit, starting at PC0. Upper limits of age are PC0=44 years PC12=45 years, PC24=46 years, and PC36=47years; ᶲAge at PC0
