# Supplementary material for: Incidence of self-reported tuberculosis treatment with community-wide universal testing and treatment for HIV and tuberculosis screening in Zambia and South Africa: A planned analysis of the HPTN 071 (PopART) cluster-randomised trial
Source: PLoS Med. 2024 May 31;21(5):e1004393. doi: 10.1371/journal.pmed.1004393 (PMC11142425; doi:10.1371/journal.pmed.1004393)
Supplement: S9 Appendix — (DOCX) [file pmed.1004393.s009.docx]

**S9 Appendix**

|  |  | **PC0** | **PC12** | **PC24** | **PC36** |
| --- | --- | --- | --- | --- | --- |
|  |  | **N=38,474** | **N=25,290** | **N=21,678** | **N=20,422** |
|  |  |  |  |  |  |
| Self-reported being told they had TB AND starting TB treatment^‡^ | Yes | 361/38474  (0.94%) | 164/25290 (0.65%) | 177/21678 (0.82%) | 110/20422 (0.54%) |
|  |  |  |  |  |  |
| Duration between visit date and MM/YYYY of TB treatment start^ꭍŦ^ | missing | 67/361  (18.56%) | 21/164  (12.80%) | 17/177 (9.6%) | 12/110 (10.91%) |
|  | ≤14 months* | 279/361  (77.29%) | 121/164  (73.78%) | 143/177 (80.8%) | 93/110 (84.55%) |
|  | >14 months* | 15/361  (4.16%) | 22/164  (13.41%) | 17/177 (9.6%) | 5/110 (4.54%) |
|  |  |  |  |  |  |
| Self-reported being told they had TB AND starting TB treatment AND duration between visit date and MM/YYYY of TB treatment start ≤14 months: overall and by country | Yes (both countries) | 279/38474  (0.73%) | 121/25290 (0.48%) | 143/21678 (0.66%) | 93/20422 (0.46%) |
|  | Zambia | 114/19724  (0.58%) | 30/12331 (0.24%) | 39/10927 (0.36%) | 29/10945 (0.26%) |
|  | SA | 165/18750  (0.88%) | 91/12959 (0.70%) | 104/10751 (0.97%) | 64/9477 (0.68%) |
|  |  |  |  |  |  |
| Self-reported being told they had TB AND starting TB treatment AND duration between visit date and MM/YYYY of TB treatment start ≤14 months: by HIV-status^$^ | Negative | 79/29130  (0.27%) | 37/17669 (0.21%) | 62/15294 (0.41%) | 38/15111 (0.25%) |
|  | Positive | 192/8004  (2.40%) | 69/5086 (1.36%) | 65/4579 (1.42%) | 53/4758 (1.11%) |
|  | Not determined | 8/1340  (0.60%) | 15/2535 (0.59%) | 16/1805 (0.89%) | 2/553 (0.36%) |

**Table: Number and proportion self-reporting being told they had TB and starting TB treatment with duration between visit date and MM/YYYY of TB treatment start ≤14 months, by Population Cohort visit, in the cohort enrolled at PC0 (N=38474) from all 21 HPTN 071 (PopART) communities**

TB=tuberculosis; PC=Population Cohort; MM/YYYY=month and year of TB treatment start **^‡^Question asked:** “in the last 12 months, have you been told that you have TB” with response options of yes, no and don’t know. If response was yes, **Question asked:** at PC0 ”have you started TB treatment” and at PC12-36 ”have you started TB treatment in the last 12 months” with response options of yes, no and don’t know; ^ꭍ^If response was yes to “in the last 12 months, have you been told that you have TB” AND yes to “have you started TB treatment/ have you started TB treatment in the last 12 months”, **Question asked:** “When did you start TB treatment? Please give the month and year?”; ^Ŧ^Denominator is number of people self-reporting being told they have TB in the last 12 months and starting TB treatment; ^¶^unable to calculate duration due to missing month and year of TB treatment start; *number of months between self-reported TB treatment start month and year, and, visit date; ^$^HIV-status based on laboratory testing.
