# Supplementary material for: Incidence of self-reported tuberculosis treatment with community-wide universal testing and treatment for HIV and tuberculosis screening in Zambia and South Africa: A planned analysis of the HPTN 071 (PopART) cluster-randomised trial
Source: PLoS Med. 2024 May 31;21(5):e1004393. doi: 10.1371/journal.pmed.1004393 (PMC11142425; doi:10.1371/journal.pmed.1004393)
Supplement: S10 Appendix — (DOCX) [file pmed.1004393.s010.docx]

**S10 Appendix**

|  |  | **2014** | | | | **2015** | | | | **2016** | | | | **2017/18** | | | |
| --- | --- | --- | --- | --- | --- | --- | --- | --- | --- | --- | --- | --- | --- | --- | --- | --- | --- |
|  |  | **A** | **B** | **C** | **Total** | **A** | **B** | **C** | **Total** | **A** | **B** | **C** | **Total** | **A** | **B** | **C** | **Total** |
| **Total N** |  | 12616^¶^  (33%)* | 13347^¶^  (35%)* | 12297^¶^  (32%)* | 38,260^¶^  (100%)* | 9406^¶^  (33%)* | 9720^¶^  (34%)* | 9442^¶^  (33%)* | 28,568^¶^  (100%)* | 8046^¶^  (32%)* | 8715^¶^  (35%)* | 8144^¶^  (33%)* | 24,905^¶^  (100%)* | 7042^¶^  (32%)* | 7889^¶^  (36%)* | 6866^¶^  (32%)* | 21,797^¶^  (100%) |
|  |  |  |  |  |  |  |  |  |  |  |  |  |  |  |  |  |  |
| Country | Zambia | 6465  (51%) | 6389  (48%) | 6736  (55%) | 19590  (51%) | 4734  (50%) | 4670  (48%) | 5040  (53%) | 14444  (51%) | 4094  (51%) | 4170  (48%) | 4388  (54%) | 12652  (51%) | 3689  (52%) | 3903  (49%) | 3931  (57%) | 11523  (53%) |
|  | SA | 6151  (49%) | 6958  (52%) | 5561  (45%) | 18670  (49%) | 4672  (50%) | 5050  (52%) | 4402  (47%) | 14124  (49%) | 3952  (49%) | 4545  (52%) | 3756  (46%) | 12253  (49%) | 3353  (48%) | 3986  (51%) | 2935  (43%) | 10274  (47%) |
|  |  |  |  |  |  |  |  |  |  |  |  |  |  |  |  |  |  |
| Sex | Male | 3578  (28%) | 3890  (29%) | 3655  (30%) | 11123  (29%) | 2572  (27%) | 2666  (27%) | 2715  (29%) | 7953  (28%) | 2112  (26%) | 2385  (27%) | 2315  (28%) | 6812  (27%) | 1799  (26%) | 2126  (27%) | 1901  (28%) | 5826  (27%) |
|  | Female | 9004  (72%) | 9417  (71%) | 8583  (70%) | 27004  (71%) | 6831  (73%) | 7050  (73%) | 6727  (71%) | 20608  (72%) | 5934  (74%) | 6328  (73%) | 5829  (72%) | 18091  (73%) | 5243  (74%) | 5763  (73%) | 4964  (72%) | 15970  (73%) |
|  | Missing | 34  (<1%) | 40  (<1%) | 59  (<1%) | 133  (<1%) | 3  (<1%) | 4  (<1%) | 0  (0%) | 7  (<1%) | 0  (0%) | 2  (<1%) | 0  (<1%) | 2  (<1%) | 0  (0%) | 0  (0%) | 1  (<1%) | 1  (<1%) |
|  |  |  |  |  |  |  |  |  |  |  |  |  |  |  |  |  |  |
| Age/years^‡^ | 18-24 | 5045  (40%) | 5162  (39%) | 4960  (40%) | 15167  (40%) | 3673  (39%) | 3692  (38%) | 3687  (39%) | 11052  (39%) | 3097  (39%) | 3279  (37%) | 3105  (38%) | 9481  (38%) | 2655  (38%) | 2922  (37%) | 2556  (37%) | 8133  (37%) |
|  | 25-29 | 2771  (22%) | 2867  (21%) | 2585  (21%) | 8223  (22%) | 2023  (22%) | 2014  (21%) | 1983  (21%) | 6020  (21%) | 1697  (21%) | 1739  (20%) | 1659  (20%) | 5095  (20%) | 1486  (21%) | 1578  (20%) | 1380  (20%) | 4444  (20%) |
|  | 30-34 | 2137  (17%) | 2278  (17%) | 2058  (17%) | 6473  (17%) | 1624  (17%) | 1713  (18%) | 1604  (17%) | 4941  (17%) | 1403  (17%) | 1563  (18%) | 1428  (18%) | 4394  (18%) | 1247  (18%) | 1414  (18%) | 1216  (18%) | 3877  (18%) |
|  | 35-39 | 1453  (12%) | 1705  (13%) | 1535  (13%) | 4693  (12%) | 1161  (12%) | 1299  (13%) | 1258  (13%) | 3718  (13%) | 1026  (13%) | 1196  (14%) | 1130  (14%) | 3352  (14%) | 916  (13%) | 1101  (14%) | 995  (15%) | 3012  (14%) |
|  | 40-44 | 1175  (9%) | 1295  (10%) | 1096  (9%) | 3566  (9%) | 922  (10%) | 998  (10%) | 910  (10%) | 2830  (10%) | 823  (10%) | 936  (11%) | 820  (10%) | 2579  (10%) | 737  (10%) | 874  (11%) | 716  (10%) | 2327  (11%) |
|  | missing | 35  (<1%) | 40  (<1%) | 63  (<1%) | 138  (<1%) | 3  (<1%) | 4  (<1%) | 0  (0%) | 7  (<1%) | 0  (0%) | 2  (<1%) | 2  (<1%) | 4  (<1%) | 1  (<1%) | 0  (0%) | 3  (<1%) | 4  (<1%) |
|  |  |  |  |  |  |  |  |  |  |  |  |  |  |  |  |  |  |
| HIV-status^†^ | Positive | 2071  (17%) | 2215  (17%) | 2099  (17%) | 6385  (17%) | 1884  (20%) | 1943  (20%) | 2047  (22%) | 5874  (21%) | 1707  (21%) | 1775  (20%) | 1854  (23%) | 5336  (21%) | 1620  (23%) | 1728  (22%) | 1649  (24%) | 4997  (23%) |
|  | Negative | 9745  (77%) | 10320  (77%) | 9372  (76%) | 29437  (77%) | 7047  (75%) | 7361  (76%) | 6864  (73%) | 21272  (74%) | 5908  (74%) | 6541  (75%) | 5761  (71%) | 18210  (73%) | 5163  (73%) | 5919  (75%) | 4977  (72%) | 16059  (74%) |
|  | ND | 800  (6%) | 812  (6%) | 826  (7%) | 2438  (6%) | 475  (5%) | 416  (4%) | 531  (5%) | 1422  (5%) | 431  (5%) | 399  (5%) | 529  (6%) | 1359  (6%) | 259  (4%) | 242  (3%) | 240  (4%) | 741  (3%) |

**Table: Characteristics of Population Cohort participants contributing person time to the cohort analysis from all 21 HPTN 071 (PopART) communities, by calendar year and study arm**

PC=Population Cohort; SA=South Africa; ND=not determined; All percentages rounded to the nearest whole number, where possible; ^¶^denominator for all column percentages shown in the column (unless otherwise indicated); *denominator is the total number contributing person time each year (row percentage); ^‡^age in years at PC0; ^†^HIV-status based on laboratory HIV-testing
