# Supplementary material for: Incidence of self-reported tuberculosis treatment with community-wide universal testing and treatment for HIV and tuberculosis screening in Zambia and South Africa: A planned analysis of the HPTN 071 (PopART) cluster-randomised trial
Source: PLoS Med. 2024 May 31;21(5):e1004393. doi: 10.1371/journal.pmed.1004393 (PMC11142425; doi:10.1371/journal.pmed.1004393)
Supplement: S11 Appendix — (DOCX) [file pmed.1004393.s011.docx]

**S11 Appendix**

|  | **2014** | | | | | | **2015** | | | | | | **2016** | | | | | | **2017/18** | | | | | |
| --- | --- | --- | --- | --- | --- | --- | --- | --- | --- | --- | --- | --- | --- | --- | --- | --- | --- | --- | --- | --- | --- | --- | --- | --- |
|  | **A** | | **B** | | **C** | | **A** | | **B** | | **C** | | **A** | | **B** | | **C** | | **A** | | **B** | | **C** | |
|  | **n/pyrs** | **rate** | **n/pyrs** | **rate** | **n/pyrs** | **rate** | **n/pyrs** | **rate** | **n/pyrs** | **rate** | **n/pyrs** | **rate** | **n/pyrs** | **rate** | **n/pyrs** | **rate** | **n/pyrs** | **rate** | **n/pyrs** | **rate** | **n/pyrs** | **rate** | **n/pyrs** | **rate** |
| **Total population** | | | | | | | | | | | | | | | | | | | | | | | | |
| triplet 1 | 4/909 | 0.44 | 6/840 | 0.71 | 4/1473 | 0.27 | 3/710 | 0.42 | 3/690 | 0.43 | 2/1223 | 0.16 | 0.5/632 | 0.08 | 3/610 | 0.49 | 2/1049 | 0.19 | 2/598 | 0.33 | 0.5/616 | 0.08 | 4/1147 | 0.35 |
| triplet 2 | 8/1594 | 0.50 | 2/1931 | 0.10 | 1/1395 | 0.07 | 3/1314 | 0.23 | 1/1563 | 0.06 | 4/1168 | 0.34 | 4/1098 | 0.36 | 5/1495 | 0.33 | 7/939 | 0.75 | 3/1130 | 0.27 | 2/1560 | 0.13 | 5/971 | 0.51 |
| triplet 3 | 3/1254 | 0.24 | 7/1039 | 0.67 | 12/1636 | 0.73 | 4/835 | 0.48 | 2/729 | 0.27 | 4/1108 | 0.36 | 1/752 | 0.13 | 3/685 | 0.44 | 5/950 | 0.53 | 1/730 | 0.14 | 2/670 | 0.30 | 3/972 | 0.31 |
| triplet 4 | 4/1334 | 0.30 | 4/ 1166 | 0.34 | 6/868 | 0.69 | 0.5/1082 | 0.05 | 5/806 | 0.62 | 3/772 | 0.39 | 1/1017 | 0.10 | 3/903 | 0.33 | 1/651 | 0.15 | 2/888 | 0.23 | 6/755 | 0.80 | 2/644 | 0.31 |
| triplet 5 | 7/1583 | 0.44 | 19/1894 | 1.00 | 19/1349 | 1.41 | 12/1325 | 0.91 | 8/1630 | 0.49 | 12/1216 | 0.99 | 12/1121 | 1.07 | 16/1393 | 1.15 | 12/949 | 1.26 | 2/919 | 0.22 | 6/1181 | 0.51 | 5/699 | 0.71 |
| triplet 6 | 10/1538 | 0.65 | 13/1619 | 0.80 | 8/1502 | 0.53 | 10/1354 | 0.74 | 10/1147 | 0.87 | 4/1388 | 0.29 | 9/1136 | 0.79 | 10/1114 | 0.90 | 17/1086 | 1.57 | 3/1071 | 0.28 | 10/1016 | 0.98 | 5/852 | 0.59 |
| triplet 7 | 9/1261 | 0.71 | 5/1496 | 0.33 | 3/1166 | 0.26 | 8/1245 | 0.64 | 4/1597 | 0.25 | 10/1248 | 0.80 | 4/1073 | 0.37 | 9/1444 | 0.62 | 12/1098 | 1.09 | 10/969 | 1.03 | 8/1266 | 0.63 | 11/890 | 1.24 |
| Overall* | 45/9473 | **0.44** | 56/9985 | **0.46** | 53/9389 | **0.41** | 40/7865 | **0.37** | 33/8161 | **0.33** | 39/8124 | **0.40** | 31/6827 | **0.27** | 49/7643 | **0.55** | 56/6722 | **0.59** | 23/6305 | **0.29** | 34/7064 | **0.36** | 35/6175 | **0.51** |
| **People living with HIV** | | | | | | | | | | | | | | | | | | | | | | | | |
| triplet 1 | 3/86 | 3.48 | 2/89 | 2.24 | 3/236 | 1.27 | 1/117 | 0.85 | 2/124 | 1.61 | 2/268 | 0.75 | 0.5/110 | 0.45 | 2/113 | 1.76 | 1/241 | 0.41 | 1/121 | 0.82 | 0.5/124 | 0.40 | 1/284 | 0.35 |
| triplet 2 | 3/196 | 1.53 | 2/386 | 0.52 | 1/195 | 0.51 | 2/236 | 0.85 | 1/366 | 0.27 | 2/218 | 0.92 | 2/212 | 0.94 | 2/356 | 0.56 | 3/183 | 1.64 | 1/245 | 0.41 | 2/405 | 0.49 | 4/204 | 1.96 |
| triplet 3 | 2/164 | 1.22 | 4/170 | 2.36 | 8/206 | 3.88 | 4/157 | 2.55 | 2/167 | 1.20 | 3/211 | 1.42 | 0.5/154 | 0.32 | 2/166 | 1.21 | 2/192 | 1.04 | 1/166 | 0.60 | 1/171 | 0.58 | 2/211 | 0.95 |
| triplet 4 | 3/262 | 1.14 | 1/189 | 0.53 | 5/180 | 2.77 | 0.5/289 | 0.17 | 2/187 | 1.07 | 1/222 | 0.45 | 1/293 | 0.34 | 1/230 | 0.44 | 0.5/205 | 0.24 | 1/259 | 0.39 | 2/207 | 0.97 | 2/211 | 0.95 |
| triplet 5 | 2/340 | 0.59 | 4/337 | 1.19 | 8/273 | 2.93 | 10/381 | 2.63 | 7/457 | 1.53 | 9/336 | 2.68 | 7/335 | 2.09 | 12/404 | 2.97 | 9/275 | 3.28 | 1/289 | 0.35 | 4/373 | 1.07 | 4/212 | 1.89 |
| triplet 6 | 6/261 | 2.30 | 6/255 | 2.35 | 4/343 | 1.16 | 4/ 297 | 1.35 | 5/188 | 2.65 | 4/470 | 0.85 | 6/262 | 2.29 | 6/175 | 3.42 | 14/387 | 3.62 | 2/285 | 0.70 | 6/167 | 3.59 | 5/330 | 1.52 |
| triplet 7 | 7/88 | 7.99 | 3/100 | 3.00 | 0.5/28 | 1.79 | 3/113 | 2.66 | 4/134 | 2.99 | 1/39 | 2.55 | 1/87 | 1.16 | 2/109 | 1.83 | 3/39 | 7.74 | 4/91 | 4.39 | 5/106 | 4.72 | 2/31 | 6.42 |
| Overall* | 26/1397 | **1.87** | 22/1526 | **1.43** | 29/1463 | **1.71** | 24/1590 | **1.17** | 23/1624 | **1.31** | 22/1764 | **1.14** | 17/1453 | **0.83** | 27/1554 | **1.38** | 32/1521 | **1.48** | 11/1458 | **0.70** | 20/1552 | **1.11** | 20/1483 | **1.42** |
| **People who were HIV-negative** | | | | | | | | | | | | | | | | | | | | | | | | |
| triplet 1 | 0.5/741 | 0.07 | 3/689 | 0.44 | 0.5/1148 | 0.04 | 2/567 | 0.35 | 1/542 | 0.18 | 0.5/925 | 0.05 | 0.5/497 | 0.10 | 1/476 | 0.21 | 1/794 | 0.13 | 1/462 | 0.22 | 0.5/474 | 0.11 | 3/854 | 0.35 |
| triplet 2 | 3/1292 | 0.23 | 0.5/1461 | 0.03 | 0.5/1131 | 0.04 | 0.5/995 | 0.05 | 0.5/1170 | 0.04 | 1/898 | 0.11 | 2/826 | 0.24 | 3/1109 | 0.27 | 4/714 | 0.56 | 2/850 | 0.24 | 0.5/1145 | 0.04 | 1/730 | 0.14 |
| triplet 3 | 0.5/1022 | 0.05 | 2/816 | 0.25 | 2/1281 | 0.16 | 0.5/649 | 0.08 | 0.5/549 | 0.09 | 1/828 | 0.12 | 1/572 | 0.17 | 1/509 | 0.20 | 3/709 | 0.42 | 0.5/547 | 0.09 | 1/492 | 0.20 | 1/754 | 0.13 |
| triplet 4 | 0.5/993 | 0.05 | 2/886 | 0.23 | 1/613 | 0.16 | 0.5/771 | 0.06 | 2/606 | 0.33 | 1/520 | 0.19 | 0.5/713 | 0.07 | 2/660 | 0.30 | 1/435 | 0.23 | 1/626 | 0.16 | 4/542 | 0.74 | 0.5/419 | 0.12 |
| triplet 5 | 4/1099 | 0.36 | 8/1263 | 0.63 | 7/958 | 0.73 | 1/856 | 0.12 | 1/1042 | 0.10 | 3/795 | 0.38 | 4/722 | 0.55 | 2/877 | 0.23 | 1/606 | 0.17 | 1/588 | 0.17 | 1/741 | 0.13 | 1/457 | 0.22 |
| triplet 6 | 4/1129 | 0.35 | 3/1305 | 0.23 | 2/961 | 0.21 | 6/974 | 0.62 | 5/926 | 0.54 | 0.5/814 | 0.06 | 3/810 | 0.37 | 4/900 | 0.44 | 3/598 | 0.50 | 1/746 | 0.13 | 4/837 | 0.48 | 0.5/502 | 0.10 |
| triplet 7 | 1/1115 | 0.09 | 2/1336 | 0.15 | 3/1111 | 0.27 | 5/1083 | 0.46 | 0.5/1374 | 0.04 | 9/1140 | 0.79 | 3/955 | 0.31 | 7/1288 | 0.54 | 9/1023 | 0.88 | 6/866 | 0.69 | 3/1140 | 0.26 | 9/851 | 1.06 |
| Overall* | 12/7392 | **0.12** | 20/7755 | **0.21** | 15/7202 | **0.15** | 14/5894 | **0.16** | 9/6208 | **0.12** | 15/5921 | **0.16** | 13/5093 | **0.21** | 20/5820 | **0.29** | 22/4878 | **0.34** | 12/4684 | **0.20** | 13/5371 | **0.20** | 15/4567 | **0.21** |
| **Sensitivity analysis (1) - People living with HIV** | | | | | | | | | | | | | | | | | | | | | | | | |
| triplet 1 | 4/144 | 2.78 | 2/138 | 1.45 | 4/310 | 1.29 | 1/121 | 0.83 | 2/125 | 1.60 | 2/276 | 0.72 | 0.5/115 | 0.43 | 2/118 | 1.69 | 1/245 | 0.41 | 1/121 | 0.82 | 0.5/124 | 0.40 | 1/284 | 0.35 |
| triplet 2 | 4/268 | 1.49 | 2/450 | 0.44 | 1/240 | 0.42 | 2/253 | 0.79 | 1/374 | 0.27 | 2/230 | 0.87 | 2/221 | 0.90 | 2/366 | 0.55 | 3/187 | 1.61 | 1/245 | 0.41 | 2/405 | 0.49 | 4/204 | 1.96 |
| triplet 3 | 3/219 | 1.37 | 5/208 | 2.41 | 10/306 | 3.27 | 4/161 | 2.49 | 2/169 | 1.18 | 3/219 | 1.37 | 0.5/156 | 0.32 | 2/171 | 1.17 | 2/200 | 1.00 | 1/166 | 0.60 | 1/171 | 0.58 | 2/211 | 0.95 |
| triplet 4 | 4/335 | 1.19 | 2/273 | 0.73 | 5/236 | 2.12 | 0.5/300 | 0.17 | 3/193 | 1.56 | 1/236 | 0.42 | 1/299 | 0.33 | 1/232 | 0.43 | 0.5/207 | 0.24 | 1/259 | 0.39 | 2/207 | 0.97 | 2/211 | 0.95 |
| triplet 5 | 3/458 | 0.65 | 10/526 | 1.90 | 12/361 | 3.32 | 10/398 | 2.51 | 7/472 | 1.48 | 9/351 | 2.57 | 7/341 | 2.06 | 13/419 | 3.10 | 10/283 | 3.53 | 1/289 | 0.35 | 4/373 | 1.07 | 4/212 | 1.89 |
| triplet 6 | 6/344 | 1.74 | 10/302 | 3.31 | 6/508 | 1.18 | 4/310 | 1.29 | 5/195 | 2.56 | 4/485 | 0.83 | 6/273 | 2.20 | 6/188 | 3.19 | 14/396 | 3.53 | 2/285 | 0.70 | 6/167 | 3.59 | 5/330 | 1.52 |
| triplet 7 | 8/112 | 7.11 | 3/132 | 2.27 | 0.5/31 | 1.61 | 3/115 | 2.61 | 4/141 | 2.85 | 1/42 | 2.36 | 1/91 | 1.10 | 2/111 | 1.80 | 3/41 | 7.40 | 4/91 | 4.39 | 5/106 | 4.72 | 2/31 | 6.42 |
| Overall* | 32/1881 | **1.77** | 34/2028 | **1.49** | 38/1992 | **1.57** | 24/1656 | **1.13** | 24/1668 | **1.36** | 22/1840 | **1.09** | 17/1495 | **0.80** | 28/1606 | **1.35** | 33/1559 | **1.47** | 11/1458 | **0.70** | 20/1553 | **1.11** | 20/1483 | **1.42** |
| **Sensitivity analysis (2) - HIV negative individuals** | | | | | | | | | | | | | | | | | | | | | | | | |
| triplet 1 | 0.5/741 | 0.07 | 3/689 | 0.44 | 0.5/1148 | 0.04 | 2/570 | 0.35 | 1/543 | 0.18 | 0.5/934 | 0.05 | 0.5/502 | 0.10 | 1/481 | 0.21 | 1/798 | 0.13 | 1/462 | 0.22 | 0.5/474 | 0.11 | 3/854 | 0.35 |
| triplet 2 | 3/1292 | 0.23 | 0.5/1461 | 0.03 | 0.5/1131 | 0.04 | 0.5/1012 | 0.05 | 0.5/1178 | 0.04 | 1/910 | 0.11 | 2/836 | 0.24 | 3/1119 | 0.27 | 4/718 | 0.56 | 2/850 | 0.24 | 0.5/1145 | 0.04 | 1/730 | 0.14 |
| triplet 3 | 0.5/1022 | 0.05 | 2/816 | 0.25 | 2/1281 | 0.16 | 0.5/653 | 0.08 | 0.5/551 | 0.09 | 1/836 | 0.12 | 1/573 | 0.17 | 1/514 | 0.19 | 3/717 | 0.42 | 0.5/547 | 0.09 | 1/492 | 0.20 | 1/754 | 0.13 |
| triplet 4 | 0.5/993 | 0.05 | 2/886 | 0.23 | 1/613 | 0.16 | 0.5/782 | 0.06 | 3/611 | 0.49 | 1/535 | 0.19 | 0.5/719 | 0.07 | 2/663 | 0.30 | 1/437 | 0.23 | 1/626 | 0.16 | 4/542 | 0.74 | 0.5/419 | 0.12 |
| triplet 5 | 4/1099 | 0.36 | 8/1263 | 0.63 | 7/958 | 0.73 | 1/872 | 0.11 | 1/1057 | 0.09 | 3/810 | 0.37 | 4/728 | 0.55 | 3/892 | 0.34 | 2/615 | 0.33 | 1/588 | 0.17 | 1/741 | 0.13 | 1/457 | 0.22 |
| triplet 6 | 4/1129 | 0.35 | 3/1305 | 0.23 | 2/961 | 0.21 | 6/987 | 0.61 | 5/932 | 0.54 | 0.5/829 | 0.06 | 3/821 | 0.37 | 4/913 | 0.44 | 3/606 | 0.49 | 1/746 | 0.13 | 4/837 | 0.48 | 0.5/502 | 0.10 |
| triplet 7 | 1/1115 | 0.09 | 2/1336 | 0.15 | 3/1111 | 0.27 | 5/1085 | 0.46 | 0.5/1380 | 0.04 | 9/1143 | 0.79 | 3/959 | 0.31 | 7/1290 | 0.54 | 9/1024 | 0.88 | 6/866 | 0.69 | 3/1140 | 0.26 | 9/851 | 1.06 |
| Overall* | 12/7392 | **0.12** | 20/7755 | **0.21** | 15/7202 | **0.15** | 14/5961 | **0.16** | 10/6253 | **0.13** | 15/5997 | **0.16** | 13/5136 | **0.21** | 21/5871 | **0.31** | 23/4916 | **0.37** | 12/4684 | **0.20** | 13/5371 | **0.20** | 15/4567 | **0.21** |

**Table: Incidence rate of self-reported TB treatment, by community, triplet, study arm and calendar year (2014 to 2017/18) among Population Cohort participants from all 21 HPTN 071 (PopART) communities**

n/pyrs=number self-reporting TB treatment/total person years; rate=per 100 person years; *rate calculated as the geometric mean of the cluster rates; Sensitivity analysis (1): If HIV-positive for a calendar year and HIV-status was not determined in the preceding year, HIV-status in the preceding year assumed to be positive and rates among people living with HIV determined; Sensitivity analysis (2): If HIV-positive for a calendar year and HIV-status was not determined in the preceding year, HIV-status in the preceding year assumed to be negative and rates among HIV negative individuals determined. NB: this excluded N=1560 who had a missing HIV-status in 2014 and a positive HIV-status in 2015, mainly due to having their enrolment visit in 2015 (1530/1560; 98%), where the HIV-status in 2014 was kept as missing.
