# Supplementary material for: Incidence of self-reported tuberculosis treatment with community-wide universal testing and treatment for HIV and tuberculosis screening in Zambia and South Africa: A planned analysis of the HPTN 071 (PopART) cluster-randomised trial
Source: PLoS Med. 2024 May 31;21(5):e1004393. doi: 10.1371/journal.pmed.1004393 (PMC11142425; doi:10.1371/journal.pmed.1004393)
Supplement: S12 Appendix — (DOCX) [file pmed.1004393.s012.docx]

**S12 Appendix**

|  | **PC0** | | | | | | **PC12** | | | | | | **PC24** | | | | | | **PC36** | | | | | |
| --- | --- | --- | --- | --- | --- | --- | --- | --- | --- | --- | --- | --- | --- | --- | --- | --- | --- | --- | --- | --- | --- | --- | --- | --- |
|  | **A** | | **B** | | **C** | | **A** | | **B** | | **C** | | **A** | | **B** | | **C** | | **A** | | **B** | | **C** | |
|  | **n/N** | **%** | **n/N** | **%** | **n/N** | **%** | **n/N** | **%** | **n/N** | **%** | **n/N** | **%** | **n/N** | **%** | **n/N** | **%** | **n/N** | **%** | **n/N** | **%** | **n/N** | **%** | **n/N** | **%** |
| **Total population** | | | | | | | | | | | | | | | | | | | | | | | | |
| triplet 1 | 3/1067 | 0.28 | 8/1006 | 0.80 | 6/1795 | 0.33 | 4/715 | 0.56 | 3/738 | 0.41 | 2/1262 | 0.16 | 0.5/651 | 0.08 | 3/634 | 0.47 | 4/1067 | 0.37 | 2/647 | 0.31 | 1/661 | 0.15 | 2/1073 | 0.19 |
| triplet 2 | 17/2019 | 0.84 | 5/2437 | 0.21 | 8/1724 | 0.46 | 4/1375 | 0.29 | 3/1633 | 0.18 | 5/1202 | 0.42 | 4/1116 | 0.36 | 5/1509 | 0.33 | 7/955 | 0.73 | 3/1178 | 0.25 | 3/1500 | 0.20 | 6/1013 | 0.59 |
| triplet 3 | 6/1647 | 0.36 | 13/1417 | 0.92 | 19/2194 | 0.87 | 4/842 | 0.48 | 2/742 | 0.27 | 4/1111 | 0.36 | 1/760 | 0.13 | 4/701 | 0.57 | 8/959 | 0.83 | 1/729 | 0.14 | 1/704 | 0.14 | 2/926 | 0.22 |
| triplet 4 | 8/1767 | 0.45 | 9/1573 | 0.57 | 12/1078 | 1.11 | 0.5/1135 | 0.04 | 2/780 | 0.26 | 4/796 | 0.5 | 1/1003 | 0.10 | 6/916 | 0.66 | 3/656 | 0.46 | 2/985 | 0.2 | 8/855 | 0.94 | 3/674 | 0.45 |
| triplet 5 | 14/2226 | 0.63 | 25/2533 | 0.99 | 40/1907 | 2.10 | 14/1389 | 1.01 | 11/1719 | 0.64 | 16/1329 | 1.2 | 14/1131 | 1.24 | 13/1454 | 0.89 | 16/958 | 1.67 | 2/994 | 0.2 | 10/1249 | 0.80 | 5/801 | 0.62 |
| triplet 6 | 18/2208 | 0.82 | 15/2352 | 0.64 | 25/2120 | 1.18 | 10/1453 | 0.69 | 15/1264 | 1.19 | 7/1477 | 0.47 | 10/1152 | 0.87 | 11/1158 | 0.95 | 14/1130 | 1.24 | 3/1100 | 0.27 | 13/1148 | 1.13 | 7/909 | 0.77 |
| triplet 7 | 11/1737 | 0.63 | 7/2086 | 0.34 | 10/1581 | 0.63 | 12/1325 | 0.91 | 9/1696 | 0.53 | 11/1307 | 0.84 | 7/1125 | 0.62 | 13/1501 | 0.87 | 16/1142 | 1.40 | 9/990 | 0.91 | 10/1299 | 0.77 | 12/987 | 1.22 |
| Overall* | 77/12671 | **0.53** | 82/13404 | **0.57** | 120/12399 | **0.81** | 48/8234 | **0.41** | 45/8572 | **0.41** | 49/8484 | **0.48** | 37/6938 | **0.31** | 55/7873 | **0.64** | 68/6867 | **0.84** | 22/6623 | **0.27** | 46/7416 | **0.43** | 37/6383 | **0.49** |
| **People living with HIV** | | | | | | | | | | | | | | | | | | | | | | | | |
| triplet 1 | 3/166 | 1.81 | 3/165 | 1.82 | 6/387 | 1.55 | 2/124 | 1.61 | 2/129 | 1.55 | 2/282 | 0.71 | 0.5/114 | 0.44 | 2/123 | 1.63 | 2/256 | 0.78 | 1/133 | 0.75 | 1/135 | 0.74 | 0.5/270 | 0.19 |
| triplet 2 | 10/338 | 2.96 | 4/559 | 0.72 | 7/304 | 2.30 | 1/257 | 0.39 | 3/385 | 0.78 | 2/225 | 0.89 | 3/217 | 1.38 | 2/371 | 0.54 | 3/169 | 1.78 | 1/265 | 0.38 | 3/387 | 0.78 | 4/217 | 1.84 |
| triplet 3 | 6/296 | 2.03 | 10/297 | 3.37 | 14/412 | 3.40 | 4/159 | 2.52 | 2/179 | 1.12 | 2/200 | 1.00 | 0.5/158 | 0.32 | 2/182 | 1.10 | 3/197 | 1.52 | 1/171 | 0.58 | 1/191 | 0.52 | 2/211 | 0.95 |
| triplet 4 | 8/454 | 1.76 | 6/375 | 1.60 | 10/292 | 3.42 | 0.5/320 | 0.16 | 1/189 | 0.53 | 2/242 | 0.83 | 1/299 | 0.33 | 3/241 | 1.24 | 2/216 | 0.93 | 1/293 | 0.34 | 3/237 | 1.27 | 3/222 | 1.35 |
| triplet 5 | 10/648 | 1.54 | 17/678 | 2.51 | 24/516 | 4.65 | 10/384 | 2.60 | 8/440 | 1.82 | 10/325 | 3.08 | 9/320 | 2.81 | 7/389 | 1.80 | 8/271 | 2.95 | 1/312 | 0.32 | 7/381 | 1.84 | 5/256 | 1.95 |
| triplet 6 | 11/501 | 2.20 | 11/460 | 2.39 | 20/730 | 2.74 | 5/313 | 1.60 | 10/216 | 4.63 | 6/449 | 1.34 | 6/257 | 2.33 | 6/188 | 3.19 | 8/364 | 2.20 | 2/284 | 0.70 | 7/197 | 3.55 | 6/357 | 1.68 |
| triplet 7 | 7/180 | 3.89 | 5/200 | 2.50 | 0.5/46 | 1.09 | 5/104 | 4.81 | 5/122 | 4.10 | 2/42 | 4.76 | 2/94 | 2.13 | 4/114 | 3.51 | 2/39 | 5.13 | 3/91 | 3.30 | 6/109 | 5.50 | 2/39 | 5.13 |
| Overall* | 55/2583 | **2.20** | 56/2734 | **1.95** | 81/2687 | **2.48** | 27/1661 | **1.26** | 31/1660 | **1.58** | 26/1765 | **1.40** | 21/1459 | **0.98** | 26/1608 | **1.58** | 28/1512 | **1.82** | 10/1549 | **0.63** | 28/1637 | **1.45** | 22/1572 | **1.33** |
| **People who were HIV-negative** | | | | | | | | | | | | | | | | | | | | | | | | |
| triplet 1 | 0.5/845 | 0.06 | 3/811 | 0.37 | 0.5/1382 | 0.04 | 2/540 | 0.37 | 1/549 | 0.18 | 0.5/926 | 0.05 | 0.5/510 | 0.10 | 1/487 | 0.21 | 2/790 | 0.25 | 1/497 | 0.20 | 0.5/503 | 0.10 | 2/793 | 0.25 |
| triplet 2 | 5/1603 | 0.31 | 1/1833 | 0.05 | 1/1373 | 0.07 | 2/962 | 0.21 | 0.5/1200 | 0.04 | 2/876 | 0.23 | 1/776 | 0.13 | 3/1087 | 0.28 | 3/653 | 0.46 | 2/874 | 0.23 | 0.5/1101 | 0.05 | 2/751 | 0.27 |
| triplet 3 | 0.5/1323 | 0.04 | 2/1088 | 0.18 | 5/1687 | 0.30 | 0.5/626 | 0.08 | 0.5/544 | 0.09 | 1/737 | 0.14 | 1/559 | 0.18 | 2/512 | 0.39 | 4/666 | 0.60 | 0.5/540 | 0.09 | 0.5/504 | 0.10 | 0.5/710 | 0.07 |
| triplet 4 | 0.5/1299 | 0.04 | 3/1177 | 0.25 | 2/747 | 0.27 | 0.5/794 | 0.06 | 1/569 | 0.18 | 1/519 | 0.19 | 0.5/698 | 0.07 | 3/658 | 0.46 | 0.5/428 | 0.12 | 1/689 | 0.15 | 5/613 | 0.82 | 0.5/433 | 0.12 |
| triplet 5 | 4/1520 | 0.26 | 7/1642 | 0.43 | 15/1330 | 1.13 | 2/841 | 0.24 | 2/970 | 0.21 | 3/754 | 0.40 | 4/690 | 0.58 | 2/819 | 0.24 | 3/553 | 0.54 | 1/629 | 0.16 | 1/765 | 0.13 | 0.5/504 | 0.10 |
| triplet 6 | 7/1512 | 0.46 | 4/1864 | 0.21 | 4/1318 | 0.30 | 4/932 | 0.43 | 5/975 | 0.51 | 0.5/752 | 0.07 | 4/746 | 0.54 | 5/883 | 0.57 | 2/558 | 0.36 | 1/760 | 0.13 | 6/933 | 0.64 | 1/519 | 0.19 |
| triplet 7 | 4/1492 | 0.27 | 2/1820 | 0.11 | 10/1464 | 0.68 | 5/1086 | 0.46 | 3/1403 | 0.21 | 9/1114 | 0.81 | 5/952 | 0.53 | 8/1245 | 0.64 | 14/1024 | 1.37 | 6/884 | 0.68 | 4/1168 | 0.34 | 10/941 | 1.06 |
| Overall* | 20/9594 | **0.14** | 22/10235 | **0.19** | 37/9301 | **0.24** | 15/5781 | **0.21** | 12/6210 | **0.16** | 16/5678 | **0.18** | 15/4931 | **0.22** | 24/5691 | **0.37** | 28/4672 | **0.42** | 12/4873 | **0.19** | 16/5587 | **0.19** | 15/4651 | **0.20** |

**Table: Proportion self-reporting TB treatment, by community, triplet, study arm and Population Cohort visit among Population Cohort participants from all 21 HPTN 071 (PopART) communities**

PC=Population Cohort; n/N=number self-reporting TB treatment/total number seen at each PC-visit; %=proportion; *proportion calculated as the geometric mean of the cluster proportion
