# Supplementary material for: Incidence of self-reported tuberculosis treatment with community-wide universal testing and treatment for HIV and tuberculosis screening in Zambia and South Africa: A planned analysis of the HPTN 071 (PopART) cluster-randomised trial
Source: PLoS Med. 2024 May 31;21(5):e1004393. doi: 10.1371/journal.pmed.1004393 (PMC11142425; doi:10.1371/journal.pmed.1004393)
Supplement: S13 Appendix. Table — (DOCX) [file pmed.1004393.s013.docx]

**S13 Appendix**

| **PC visit** | **accounting for between-trial-arm variation** | **accounting for between-trial-arm variation and between-triplet variation** |
| --- | --- | --- |
| **PC0** | 0.48 | 0.42 |
| **PC12** | 0.50 | 0.22 |
| **PC24** | 0.44 | 0.00 |
| **PC36** | 0.52 | 0.18 |

**Table: The estimated coefficient of between-community variation k at each Population Cohort visit (PC0, PC12, PC24 and PC36, respectively).**

PC=population cohort;
