# Supplementary material for: Incidence of self-reported tuberculosis treatment with community-wide universal testing and treatment for HIV and tuberculosis screening in Zambia and South Africa: A planned analysis of the HPTN 071 (PopART) cluster-randomised trial
Source: PLoS Med. 2024 May 31;21(5):e1004393. doi: 10.1371/journal.pmed.1004393 (PMC11142425; doi:10.1371/journal.pmed.1004393)
Supplement: S1 CONSORT Checklist — (DOCX) [file pmed.1004393.s014.docx]

CONSORT 2010 checklist of information to include when reporting a randomised trial, with extension to cluster randomized trials checklist items (*shown in italics*) taken from BMJ 2012;345:e5661

| **Section/Topic** | **Item No** | **Checklist item** | **Reported on*** |
| --- | --- | --- | --- |
| **Title and abstract** | 1a | Identification as a *cluster* randomised trial in the title | Included in the title |
|  | 1b | Structured summary of trial design, methods, results, and conclusions _(for_ _specific_ _guidance_ _see_ _CONSORT_ _for_ _abstracts)_ | Structured abstract included |
| **Introduction**  Background and | 2a | Scientific background, explanation of rationale and *rationale for using a cluster design* | Background  paragraph 1-5 |
| objectives | 2b | Specific objectives or hypotheses and *whether objectives pertain to the cluster level, the individual participant level of both* | Background  paragraph 5 |
| **Methods**  Trial design | 3a | Description of trial design (such as parallel, factorial) including allocation ratio; *definition of cluster and description of how the design features apply to the clusters* | Methods paragraph 2 |
|  | 3b | Important changes to methods after trial commencement (such as eligibility criteria), with reasons | n/a |
| Participants | 4a | Eligibility criteria for participants; *eligibility criteria for clusters* | Methods paragraph 2 |
|  | 4b | Settings and locations where the data were collected | Methods paragraph 2 |
| Interventions | 5 | The interventions for each group with sufficient details to allow replication, including how and when they were actually administered; *whether the intervention pertain to the cluster level, the individual participant level, or both* | Methods paragraph 3 |
| Outcomes | 6a | Completely defined pre-specified primary and secondary outcome measures, including how and when they were assessed; *whether outcome measures pertain to the cluster level, the individual participant level, or both* | Methods paragraph 4-5  Appendix-S4 |
|  | 6b | Any changes to trial outcomes after the trial commenced, with reasons | Appendix-S4 |
| Sample size | 7a | How sample size was determined; *method of calculation, number of clusters(s) (and whether equal or unequal cluster sizes are assumed), cluster size, a coefficient of intracluster correlation (ICC or k), and an indication of its uncertainty* | * |
|  | 7b | When applicable, explanation of any interim analyses and stopping guidelines | * |
| Randomisation: Sequence generation | 8a | Method used to generate the random allocation sequence | * |
|  | 8b | Type of randomisation; details of any restrictions (such as blocking and block size); *details of stratification or matching if used* | Methods paragraph 2 and * |
| Allocation concealment mechanism | 9 | Mechanism used to implement the random allocation sequence (such as sequentially numbered containers), describing any steps taken to conceal the sequence until interventions were assigned; *specification that allocation was based on clusters rather than individuals and whether allocation concealment (if any) was at the cluster level, the individual participant level, or both* | * |
| Implementation | 10a | Who generated the random allocation sequence, who enrolled clusters, and who assigned clusters to interventions | * |
|  | 10b | Mechanism by which individual participants were included in clusters for the purposes of the trial (such as complete enumeration, random sampling) | Methods paragraph 3 |
|  | 10c | From whom consent was sought (representatives of the cluster, or individual cluster members, or both) and whether consent was sought before or after randomisation | * |
| Blinding | 11a | If done, who was blinded after assignment to interventions (for example, participants, care providers, those assessing outcomes) and how | n/a |

|  | 11b | If relevant, description of the similarity of interventions | n/a |
| --- | --- | --- | --- |
| Statistical methods | 12a | Statistical methods used to compare groups for primary and secondary outcomes; *how clustering was into account* | Methods paragraph 6-12  Appendix-S4 |
|  | 12b | Methods for additional analyses, such as subgroup analyses and adjusted analyses | Methods paragraph 12  Appendix-S4 |
| **Results**  Participant flow (a diagram is strongly | 13a | For each group, the numbers of participants who were randomly assigned, received intended treatment, and were analysed for the primary outcome; *for each group, the numbers of clusters that were randomly assigned, received intended treatment, and were analysed for the primary outcome* | Figure 1 |
|  | 13b | For each group, losses and exclusions after randomisation, together with reasons; *for each group, losses and exclusions for both clusters and individual cluster members* | Figure 1 |
| Recruitment | 14a | Dates defining the periods of recruitment and follow-up | Defined in the methods (paragraph 3 and 4) |
|  | 14b | Why the trial ended or was stopped | Defined in the methods (paragraph 3 and 4) |
| Baseline data | 15 | A table showing baseline demographic and clinical characteristics for each group; *baseline characteristics for the individual and cluster levels as applicable for each group* | Results Table 2 |
| Numbers analysed | 16 | For each group, number of participants (denominator) included in each analysis and whether the analysis was by original assigned groups; *for each group, number of clusters included in each analysis* | Results paragraph 1-2 |
| Outcomes and estimation | 17a | For each primary and secondary outcome, results for each group, and the estimated effect size and its precision (such as 95% confidence interval); *results at the individual or cluster level as applicable*  *and a coefficient of intracluster correlation (ICC or k) for each primary outcome* | Results paragraph 3-6 |
|  | 17b | For binary outcomes, presentation of both absolute and relative effect sizes is recommended | Results paragraph 3-6 |
| Ancillary analyses | 18 | Results of any other analyses performed, including subgroup analyses and adjusted analyses, distinguishing pre-specified from exploratory | Results paragraph 4 and 6 |
| Harms | 19 | All important harms or unintended effects in each group | n/a |
| **Discussion**  Limitations | 20 | Trial limitations, addressing sources of potential bias, imprecision, and, if relevant, multiplicity of analyses | Discussion paragraph 4 and 8 |
| Generalisability | 21 | Generalisability (external validity, applicability) of the trial findings; *generalisability to clusters and/or individual participants (as relevant)* | Discussion paragraph 1, 5 and 6 |
| Interpretation | 22 | Interpretation consistent with results, balancing benefits and harms, and considering other relevant evidence | Discussion paragraph 1-3, 6-7 |
| **Other information**  Registration | 23 | Registration number and name of trial registry | * |
| Protocol | 24 | Where the full trial protocol can be accessed, if available | * |
| Funding | 25 | Sources of funding and other support (such as supply of drugs), role of funders | Funding statement provided |

*Please note that some items have been marked as see additional information because the information has been reported elsewhere. The current manuscript is not the main results of the HPTN 071 trial (which has been published, is referred to and referenced throughout the manuscript). The following provides additional information about the parent trial.

HPTN 071 study overview and trial protocol versions: <https://www.hptn.org/research/studies/hptn071#block-views-block-study-related-publications-block-1>

HPTN 071 trial protocol: doi: 10.1186/1745-6215-15-57

HPTN 071 trial primary outcome paper: DOI: 10.1056/NEJMoa1814556

TB reduction through ART and TB screening (TREATS) project – which aimed to measure TB outcomes of HPTN 071, study overview: clinicaltrials.gov ID NCT03739736
